# PDGFR-modulated miR-23b cluster and miR-125a-5p suppress lung tumorigenesis by targeting multiple components of KRAS and NF-kB pathways

**DOI:** 10.1038/s41598-017-14843-6

**Published:** 2017-11-13

**Authors:** Srivatsava Naidu, Lei Shi, Peter Magee, Justin D. Middleton, Alessandro Laganá, Sudhakar Sahoo, Hui Sun Leong, Melanie Galvin, Kristopher Frese, Caroline Dive, Vincenza Guzzardo, Matteo Fassan, Michela Garofalo

**Affiliations:** 1 Transcriptional Networks in Lung Cancer Group, Cancer Research UK Manchester Institute, University of Manchester, Manchester, UK; 2 Department of Molecular Virology, Immunology and Medical Genetics, Comprehensive Cancer Center, The Ohio State University, Columbus, USA; 3 Department of Genetics and Genomic Sciences, Icahn School of Medicine at Mount Sinai, New York City, USA; 4 Computational Biology Support Group, Cancer Research UK Manchester Institute, University of Manchester, Manchester, UK; 5 Clinical and Experimental Pharmacology Group, Cancer Research UK Manchester Institute, University of Manchester, Manchester, UK; 6 Cancer Research UK Lung Cancer Centre of Excellence, at Manchester and University College London, London, UK; 7 Department of Medicine, Surgical Pathology Unit, University of Padua, Padua, Italy

## Abstract

In NSCLC alterations in PDGF receptors are markers of worst prognosis and efficient targeting of these receptors is yet to be achieved. In this study, we explored PDGFR-regulated microRNAs demonstrating that miR-23b cluster and miR-125a-5p are downregulated by increased expression of PDGFR-α or PDGFR-β in NSCLC cells. Mechanistically, the expression of these microRNAs is positively regulated by p53 and negatively modulated by NF-kB p65. Forced expression of miR-23b cluster or miR-125a-5p enhanced drug sensitivity and suppressed invasiveness of NSCLC cells by silencing several genes involved in oncogenic KRAS and NF-kB pathways, including SOS1, GRB2, IQGAP1, RALA, RAF-1, IKKβ, AKT2, ERK2 and KRAS itself. Of note, an inverse correlation between miR-23b cluster, miR-125a-5p and respective target genes was also found *in vivo* in a large dataset of lung adenocarcinoma samples. Furthermore, *in vivo* delivery of miR-23b cluster or miR-125a-5p significantly repressed tumour growth in a highly aggressive NSCLC circulating tumour cell (CTC) patient derived explant (CDX) mouse model. In conclusion, our finding sheds light on the PDGFR signaling and endorses the possibility to employ miR-23b cluster and miR-125a-5p as therapeutic tools to silence simultaneously a range of redundant pathways and main effectors of tumorigenesis in NSCLC.

## Introduction

Lung cancer ranks first in cancer morbidity and mortality rates globally^1,2^. The most frequently diagnosed histological sub-type, non-small cell lung cancer (NSCLC), accounts for 80–85% of cases, with a disappointing 5 year survival rate of 17.4%^3^. During the past several years effective targeted therapies have been delivered, including erlotinib, gefitinib and most recently osimertinib and crizotinib/ceritinib for patients harboring EGFR activating mutations and ALK/EML4 translocations, respectively. However, EGFR*-*mutant and ALK*-*rearranged cancers constitute less than one-fifth of all NSCLC cases and patients that initially respond well to therapy inevitably relapse few months later^4^. Thus, identification of other potential molecular targets and novel therapeutic approaches is of utmost importance. It is now accepted that NSCLC is not a singular entity but a heterogeneous disease and optimal management of NSCLC requires targeted therapies^5^. For the development of targeted therapies, PDGF receptors and their ligands, platelet-derived growth factors (PDGFs), are attractive candidates due to their effect on cellular proliferation, migration and survival^6^. There are five different isoforms of PDGF that activate cellular response through receptors alpha (PDGFR-α) and beta (PDGFR-β). Both PDGF ligands and the receptors have been detected in lung cancer cells but not in normal cells and are markers of worse prognosis^7^. Several tyrosine kinase inhibitors, including imatinib, have been developed to block PDGFRs, however they are not selective and inhibit also other kinases^8^. Furthermore, these inhibitors did not show significant effects *in vivo* ^9^. PDGF receptors exert their oncogenic function by activating the oncogene KRAS and three major survival pathways: Raf/Mek/Erk, PI3K/Akt and the Ral guanine nucleotide exchange factors (Ral-GEFs)^10–12^. Direct inhibition of KRAS is currently unavailable in the clinic and therapeutic strategies targeting KRAS indirectly have so far largely been futile due to the activation of cellular compensatory mechanisms^13^. MicroRNAs (miRNAs) are endogenous small non-coding RNAs (~22 nucleotides) implicated in the regulation of fundamental cellular processes^14^. Deranged miRNA expression is reported as a cause or consequence of various human malignancies, including lung cancer, and emerging evidence suggests that microRNAs could be potential therapeutic tools for cancer management especially in combination with anti-cancer drugs^15^. The striking advantage of miRNAs as therapeutics is that a single microRNA, by targeting multiple genes, has the potential to simultaneously dampen several oncogenic pathways. This could minimise requirement of drug combinations with their increased potential for toxic side effects and activation of drug-induced compensatory mechanisms as well development of acquired resistance^16^. In this study, we identified miR-23b-miR-27b-miR-24-1 (referred as miR-23b cluster) and miR-125a-5p as PDGFR-modulated microRNAs. Enforced expression of miR-23b cluster and miR-125a-5p silenced directly or indirectly multiple genes involved in the KRAS and NF-kB signaling reducing cell proliferation and enhanced drug-induced apoptosis. We demonstrated that miR-23b cluster and miR-125a-5p are transcriptionally activated by p53 and negatively regulated by NF-kB p65 transcription factors. *In vivo* delivery of these microRNAs suppressed the growth of a highly aggressive tumor derived from a patient with metastatic NSCLC and unresponsive to standard-of-care chemotherapy. These results shed new light on the mechanisms involved in lung tumorigenesis and lay the foundations to potentially develop more effective therapeutic strategies for both PDGFR- and KRAS-driven NSCLC.

## Results

### PDGFR-modulated microRNAs

**Figure 1:**
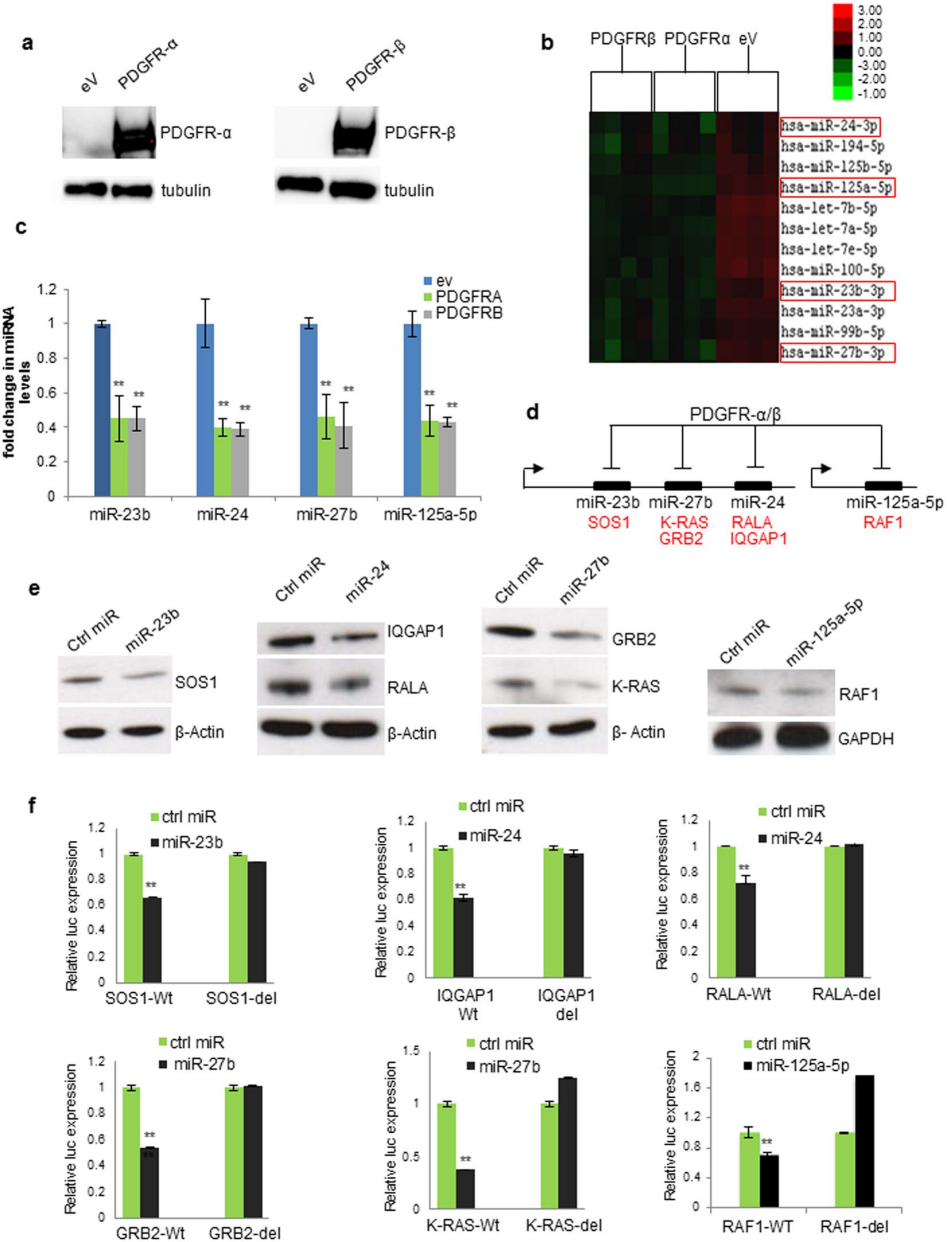
PDGFR-α- and PDGFRβ-regulated miRNAs. (**a**) Overexpression of PDGFR-α or PDGFR-β in A549 cells. (**b**) Unsupervised hierarchical clustering of miRNAs expression after PDGFR-α or PDGFR-β overexpression compared to empty vector (eV). P values were obtained by ANOVA test (*p* < 0.05). Complete heatmap is reported in Supplementary Fig. 1SA. (**c**) Downregulation of miR-23b, miR-24, miR-27b and miR-125a-5p after PDGFR-α or PDGFR-β overexpression. (**d**) Schematic representation of downregulated miRNAs after PDGFR-α or PDGFR-β overexpression and respective putative target genes. (**e**) Western blots showing downregulation of target genes. GAPDH or β-actin was used as loading control. (**f**) SOS1, IQGAP1, RALA, GRB2, KRAS and RAF-1 3′UTRs are direct targets of miR-23b, miR-24, miR-27b and miR-125a-5p. pGL3-SOS1, pGL3-IQGAP1, pGL3-RALA, pGL3-Grb2,pGL3-KRAS, pGL3-Raf-1 reporter vectors, containing Wt or mutated (del) 3′UTRs were transfected into HEK293 cells. Results are from at least three independent experiments. Uncropped western blots are available in the Supplementary information. Error bars indicate mean ± SD. (n = 3). **p < 0.01 by two-tailed Student’s t test. Ctrl = control.

To investigate the molecular mechanisms involved in PDGFR-driven lung tumourigenesis we overexpressed PDGFR-α or PDGFR-β in A549 cells (Fig. 1a) and analysed the global miRNA expression profile using the NanoString technology. Clustering analysis revealed differential miRNA levels in PDGFR-α- or PDGFR-β-overexpressing data sets compared to controls (*p* < 0.05, Fig. 1b; Supplementary Fig. S1A). Since PDGFRs are well-known oncogenes, we have filtered the miRNAs that were down-modulated after PDGFRs overexpression, and hypothesised that the suppressed miRNA subset may potentially have tumour suppressor role(s). MiR-23b cluster and miR-125a-5p were commonly and significantly downregulated after PDGFRs overexpression (Fig. 1b; Supplementary Fig. S1A) as confirmed by quantitative real-time PCR (qPCR) (Fig. 1c). Importantly, PDGFR-β silencing in H1703 cells or treatment with imatinib gave rise to an increase in the expression of these miRNAs, confirming that also in physiological conditions miR-23b cluster and miR-125a-5p are regulated by PDGFRs (Supplementary Fig. S1B). It is known that PDGFRs induce tumorigenesis via RAS activation^17^. Interestingly, target prediction revealed that KRAS pathway components such as SOS1, GRB2, RALA, IQGAP1, RAF1 and KRAS itself, were putative target genes of these miRNAs (Fig. 1d; Supplementary Fig. S2A). Forced expression of miR-23b cluster or miR-125a-5p by synthetic microRNA mimics markedly reduced the protein levels encoded by the respective target genes as shown in Fig. 1e. Furthermore, to verify a possible direct interaction, the 3′ UTRs of target genes were cloned into the pGL3 control reporter vector downstream of the luciferase gene (Supplementary Fig. S2B). Co-transfection of pGL3-3′ UTR constructs along with the corresponding miRNA significantly reduced the relative luciferase activity. This repression was rescued by the deletion of the miRNA binding sites (Fig. 1f; Supplementary Fig. S2B). PDGFR-α or PDGFR-β overexpression increased endogenous levels of KRAS, IQGAP1, RALA and RAF-1 (Supplementary Fig. S3A), whereas coexpression of PDGFR-β and miR-24/miR-27b rescued this effect (Supplementary Fig. S3B). These results corroborate the hypothesis that PDGFRs induce upregulation of oncogenes involved in the KRAS pathway through the silencing of miR-23b cluster and miR-125a-5p.

### MiR-23b cluster and miR-125a-5p suppress AKT and ERKs signaling

**Figure 2:**
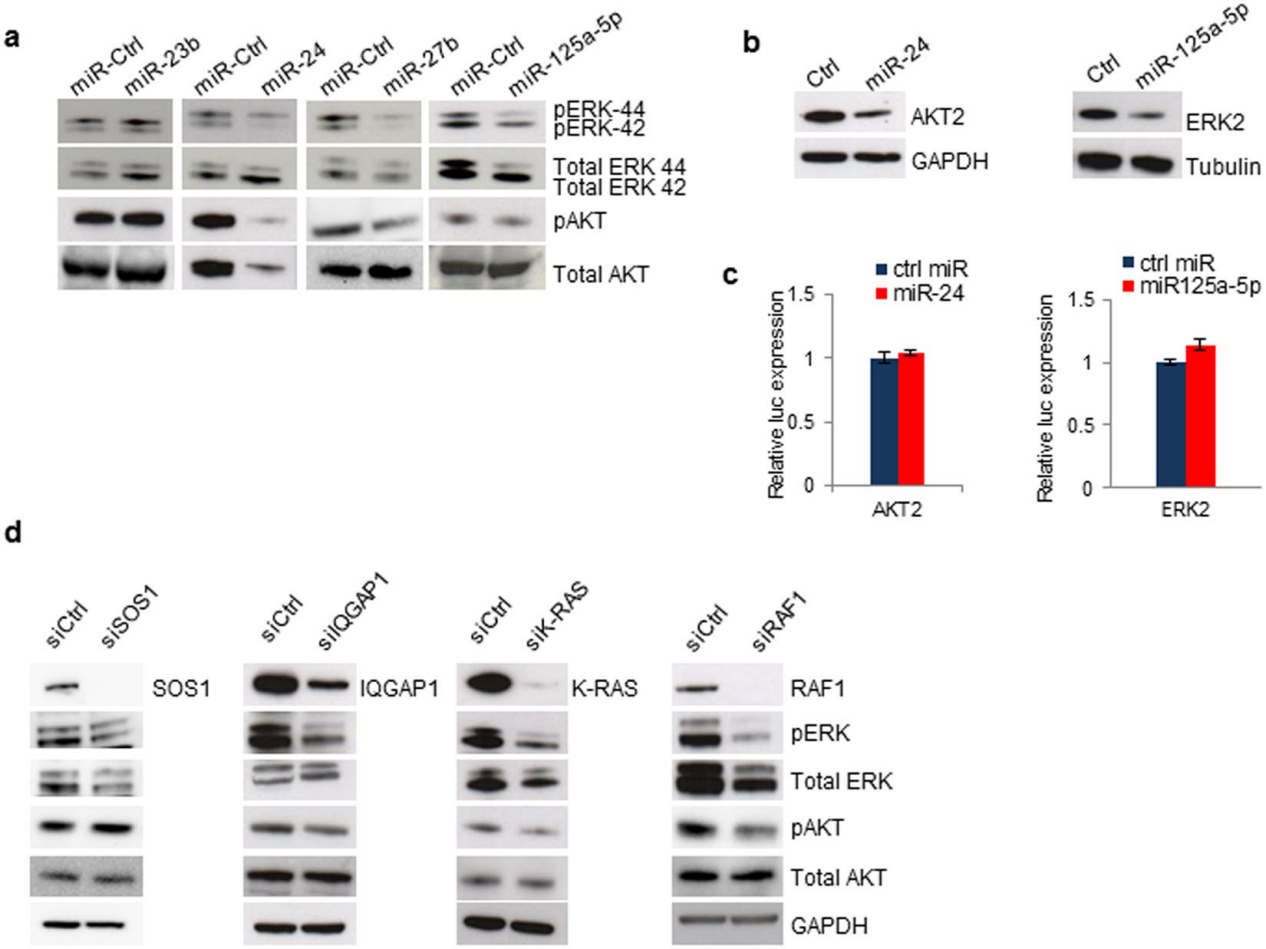
MiR-23b cluster and miR-125a-5p modulate ERKs/AKT signaling. (**a**) Western blots showing the effect of miR-23b, miR-24, miR-27b and miR-125a-5p enforced expression on ERKs or AKT activation. (**b)** AKT2 and ERK2 silencing after miR-24 or miR-125a-5p overexpression. (**c**) Luciferase assay showing indirect regulation of AKT2 and ERK2 after miR-24 or miR125a-5p overexpression. (**d**) Effect of siRNA mediated knockdown of SOS1, IQGAP1, KRAS and RAF-1 on ERKs and AKT pathways in A549 cells. Uncropped western blots are available in the Supplementary information.

KRAS transduces multiple downstream oncogenic signals that would ultimately lead to cell survival and proliferation^10–12^. Since miR-23b cluster and miR-125a-5p silenced the expression of KRAS signaling components, we investigated whether these miRNAs could modulate the activation of pro-survival signaling pathways such as ERKs and AKT. Immunoblotting analysis revealed that while enforced expression of miR-23b did not affect ERKs or AKT phosphorylation levels, miR-24, miR-27b and miR-125a-5p reduced both ERK and AKT activation in A549 cells (Fig. 2a). Endogenous AKT and ERK2 levels were also markedly downregulated after miR-24 or miR-125a-5p transfection, respectively. Subsequent target prediction identified AKT2 and ERK2 as putative targets of the respective miRNAs, which was confirmed by immunoblot analysis (Fig. 2b). However, reporter gene expression of 3′ UTR constructs was not significantly altered after miRNAs transfection, suggesting an indirect regulation (Fig. 2c). In line with these results, siRNA mediated knockdown of IQGAP1, KRAS and RAF1, but not SOS1 expression, resulted in a similar dampening effect of ERK or AKT phosphorylation that was observed after the corresponding miRNA enforced expression (Fig. 2d). Taken together, these results indicate that miR-24, miR-27b and miR-125a-5p directly and indirectly suppress AKT and ERKs activation in NSCLC.

### MiR-23b cluster and miR-125a-5p enhance drug sensitivity in NSCLC cell lines

**Figure 3:**
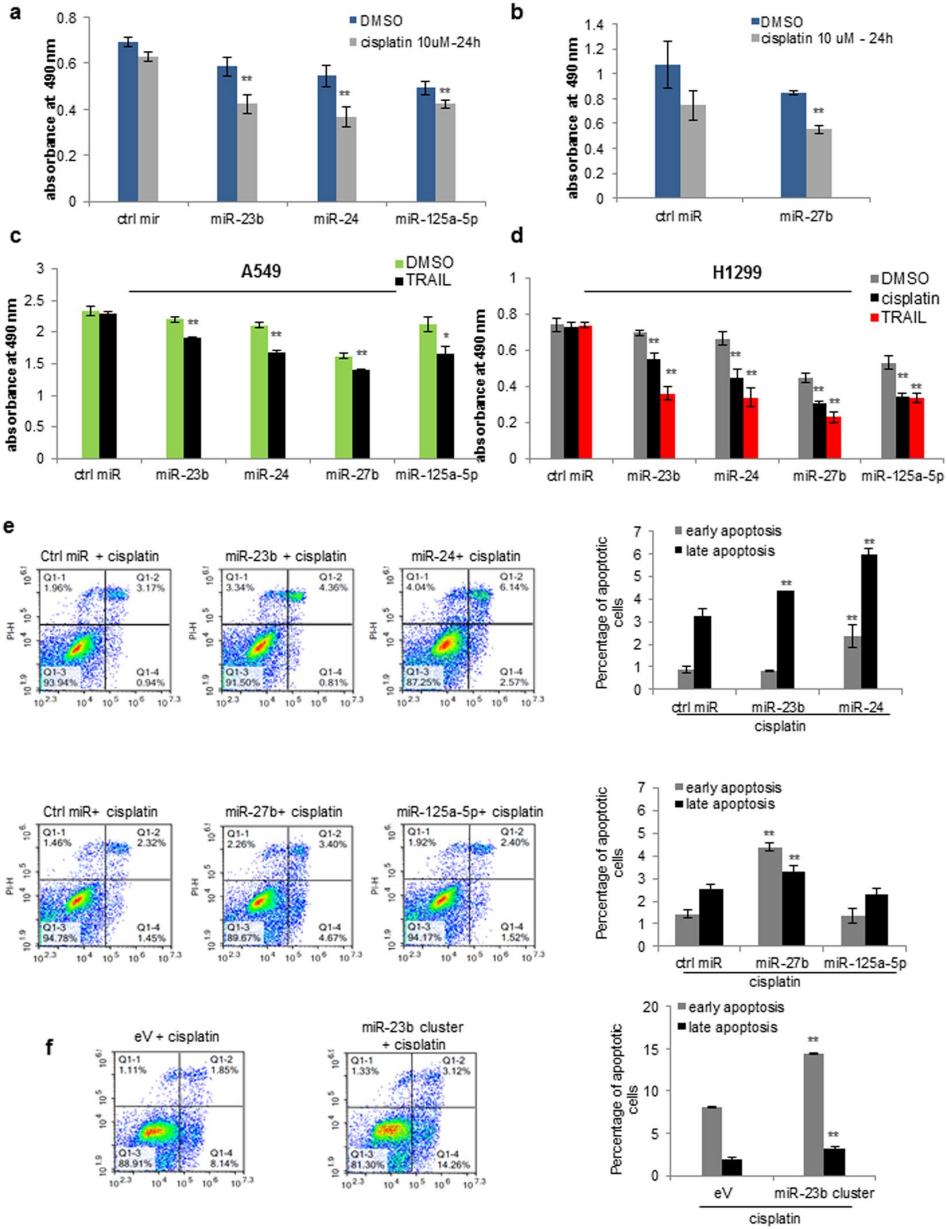
MiR-23b cluster and miR-125a-5p induce drug sensitivity in NSCLC cell lines. (**a,b**) Enforced expression of miR-23b, miR-24, miR-27b and miR-125a-5p in A549 cells reduced cell proliferation after cisplatin treatment. (**c,d**) Enforced expression of miR-23b, miR-24, miR-27b and miR-125a-5p in A549 and H1299 cells decreased cell proliferation after cisplatin or TRAIL treatment. (**e**) Overexpression of miR-23b, miR-24 and miR-27b increased apoptosis after cisplatin treatment in A549 cells, whereas miR-125a-5p had no effect. (**f**) Cells stably expressing miR-23b cluster are more sensitive to cisplatin compared to control cells, as assessed by Annexin V. Error bars indicate mean ± SD (n = 3). *p < 0.05, **p < 0.01 by two-tailed Student’s t test.

Activated ERK/AKT signaling can contribute to drug resistance in cancer cells^18^. Given the suppressive role of miR-24, miR-27b and miR-125a-5p on ERKs/AKT activation, we examined the effect after overexpression of these miRNAs in response to pemetrexed alone or as platinum doublet, commonly used as first line chemotherapy in patients with advanced NSCLC^19^ or TRAIL. As confirmed by proliferation assay, A549 cells transfected with miR-23b, miR-24, miR-27b or miR-125a-5p were significantly less viable after cisplatin, pemetrexed (or the combination cisplatin/pemetrexed) and after TRAIL treatment compared to control cells (*p* < 0.001, Fig. 3a–c, Supplementary Fig. 4a). Also, reconstitution of miR-23b cluster or miR-125a-5p in H1299 increased sensitivity to cisplatin or TRAIL treatment (Fig. 3d). As expected, RALA, KRAS, RAF1 and IQGAP1 silencing resulted in significantly less viable cells in response to cisplatin when compared to control (Supplementary Fig. S4B). Next, we analysed the effect of these miRNAs on cisplatin-induced apoptosis using AnnexinV-FITC/PI staining followed by flow cytometry analysis. Enforced expression of miR-23b, miR-24 and miR-27b but not of miR-125a-5p significantly enhanced cisplatin-induced apoptosis in A549 cells (*p* < 0.001, Fig. 3e). To better understand the extent of the effect these miRNAs exerted on apoptosis we stably overexpressed miR-23b cluster and miR-125a-5p in A549 cells and a similar trend on increased apoptosis was observed (Fig. 3f and Supplementary Fig. S4C). In summary, miR-23b cluster affects proliferation and survival whilst miR-125a-5p affects proliferation of NSCLC cells without increasing apoptosis.

### MiR-23b cluster and miR-125a-5p regulate cell cycle progression and mesenchymal-epithelial transition (MET)

**Figure 4:**
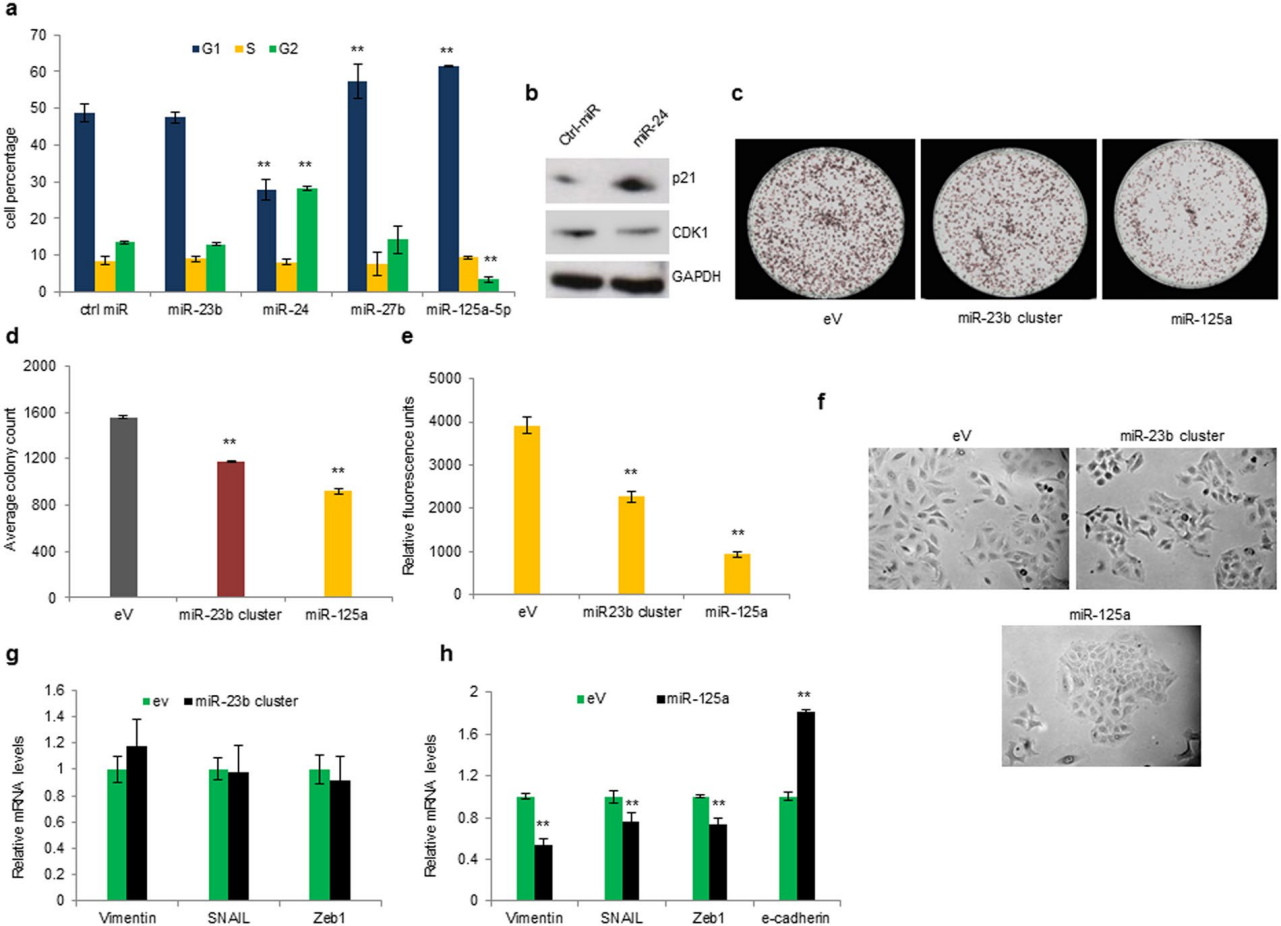
Effect of miR-23b cluster and miR-125a-5p enforced expression on cell cycle progression and MET. (**a**) Cell cycle analysis of A549 cells transiently transfected with miR-23b, miR-24, miR-27b or miR-125a-5p. MiR-23b does not affect cell cycle progression, miR-24 blocks the cells in G2 phase whereas miR-27b and miR-125a-5p arrest cell cycle in G1 phase. (**b**) Western blot showing p21 upregulation and CDK1 downregulation after miR-24 forced expression in A549 cells. (**c,d**) Colony formation assay of A549 cells stably expressing miR-23b cluster or miR-125a-5p. (**e**) MiR-23b cluster and miR-125a-5p stable cell lines are significantly less invasive compared to control cells. (**f**) Morphology of cells stably expressing miR-23b cluster or miR-125a-5p compared to control cells. (**g,h**) Epithelial and mesenchymal markers in cells expressing miR-23b cluster or miR-125a-5p compared to control cells. Uncropped western blots are available in the Supplementary information. All the experiments were performed at least in triplicates. Error bars indicate mean ± SD (n = 3). **p < 0.01 by two-tailed Student’s t test.

To further investigate the tumour suppressor role of miR-23b cluster and miR-125a-5p we analysed their effect on cell cycle progression and clonogenicity. A549 cells were transiently transfected with miR-23b, miR-24, miR-27b, miR-125a-5p or control miRNA and analysed by flow cytometry. MiR-23b did not exert any effect on cell cycle progression; however, cells transfected with miR-24 underwent G2/M arrest whereas miR-27b and miR-125a-5p overexpression caused a significant G1/S arrest compared to control cells (*p* < 0.001, Fig. 4a; Supplementary Fig. S5A). To probe the underlying mechanism, we examined the expression of p21 which plays a crucial role in G2/M arrest and CDK1 an essential regulator of G2/M transition that is negatively regulated by p21^20,21^. As expected, forced expression of miR-24 markedly increased p21 expression and caused a reduction in CDK1 levels (Fig. 4b). Thus, G2/M arrest caused by miR-24 may be attributed to elevated p21 and reduced CDK1 protein levels. Next, we tested clonogenic capacity and invasiveness of A549 cells stably expressing either miR-23b cluster or miR-125a-5p. Cells expressing either miR-23b cluster or miR-125a-5p (Supplementary Fig. S5B) showed a marked reduction in clone forming capacity and invasiveness compared to control cells (Fig. 4c–e). Furthermore, A549 cells stably overexpressing miR-125a-5p and not the miR-23b cluster, showed morphological changes consistent with a mesenchymal-epithelial transition (MET) (Fig. 4f). Analysis of epithelial and mesenchymal markers confirmed a prominent reduction of vimentin, Snail and Zeb1 in miR-125a-5p stable compared to miR-23b cluster stable and control cells (Fig. 4g,h).

### p53 transcriptionally activates miR-23b cluster and miR-125a-5p

**Figure 5:**
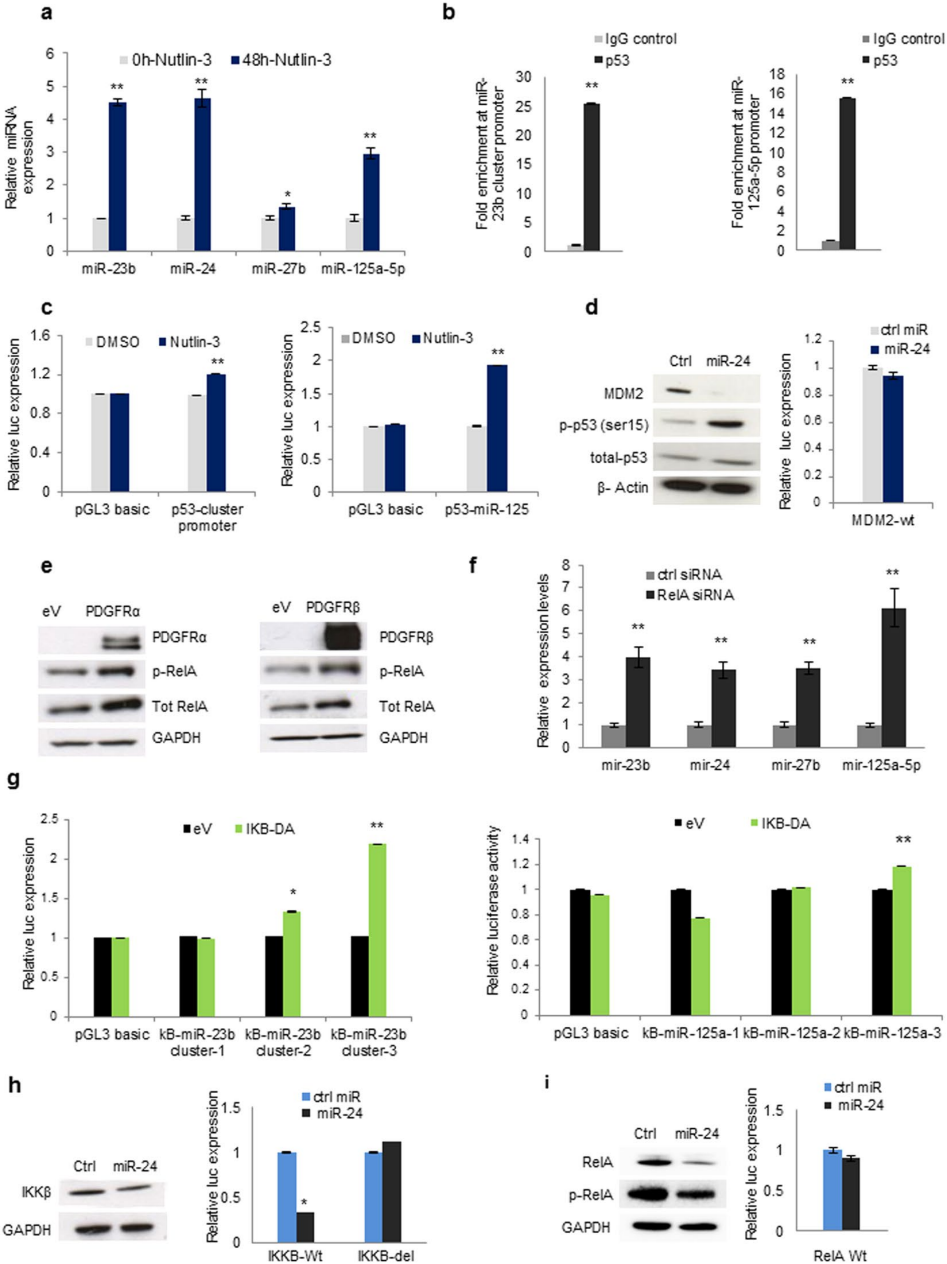
MiR-23b cluster and miR-125a-5p are transcriptionally activated by p53 and negatively regulated by NF-kB p65. (**a**) Nutlin-3 treatment induces upregulation of miR-23b cluster and miR-125a-5p. (**b**) Chromatin immunoprecipitation assay (ChIP) showing that p53 binds to miR-23b cluster and miR-125a-5p promoter regions (**c**) Promoter regions of miR-23b cluster and miR-125a-5p containing p53 binding sites were cloned into pGL3 basic reporter vector. Treatment with nutlin-3 increased luciferase activity. (**d**) MiR-24 indirectly targets MDM2 and induces p53 activation. (**e**) PDGFR-α or PDGFR-β overexpression activated NF-kB p65. (**f**) NF-kB p65 silencing induced upregulation of miR-23b cluster and miR-125a-5p in A549 cells. (**g**) MiR-23b cluster and miR-125a-5p promoter regions containing NF-kB binding sites were cloned in pGL3 basic reporter vector. Luciferase assays in A549 cells after co-transfection with reporter constructs and a dominant active NF-kB p65 inhibitor (IKKβ). (**h,i**) MiR-24 targets directly IKKβ (H) and indirectly RelA (I). Uncropped western blots are available in the Supplementary information. Error bars indicate mean ± SD (n = 3) and p values were calculated by two-tailed Student t test (*p < 0.05, **p < 0.001).

The data presented so far suggested anti-tumour properties for miR-23b cluster and miR-125a-5p, so we asked if p53, as an established tumour suppressor transcription factor, could be involved in the regulation of these microRNAs. Treatment with nutlin-3, which interferes with MDM2/p53 interaction and stabilizes p53, markedly upregulated the expression of miR-23b cluster and miR-125a-5p in A549 cells (Fig. 5a), suggesting that these miRNAs may be transcriptionally activated by p53. To further investigate the regulatory mechanism, we computationally analysed the locus upstream of the miR-23b cluster or miR-125a-5p coding sequences and found multiple putative p53 binding sites within these regions. ChIP analysis confirmed the binding of p53 at these predicted sites for both miR-23b cluster and miR-125a-5p (Fig. 5b). To verify the activity of p53 at these regions miR-23b cluster and miR-125a-5p promoter fragments containing p53 binding sites were cloned into a promoterless reporter vector. Treatment with nutlin-3 increased the reporter gene activity (Fig. 5c), confirming a functional binding of p53 to miR-23b cluster and miR-125a-5p promoter. MDM2 has computationally been predicted to be a target of miR-24. Forced expression of miR-24 significantly reduced MDM2 expression, but did not affect MDM2-3′ UTR luciferase activity suggesting that MDM2 is a miR-24 indirect target. Consequently, miR-24 dependent silencing of MDM2 increased p53 phosphorylation at serine 15 (Fig. 5d). Taken together, these results show that p53 regulates miR-23b cluster and miR-125a-5p expression. In turn, miR-24 controls p53 expression by indirectly silencing MDM2 and thereby establishing a positive feedback loop.

### NF-kB p65 regulates miR-23b cluster and miR-125a-5p expression

The oncogenic role of NF-kB p65 signaling in lung cancer has been widely reported and the importance of NF-kB p65 in PDGFR-KRAS signaling has been described previously^22,23^. To elucidate the role of NF-kB p65 in the regulation of miR-23b cluster and miR-125a-5p, we overexpressed PDGFR-α or PDGFR-β in A549 cells and checked NF-kB p65 phosphorylation and total levels. PDGFRs overexpression markedly induced not only RelA phosphorylation but also RelA total levels (Fig. 5e). Next, to verify whether NF-kB p65 could be involved in miR-23b cluster and miR-125a-5p silencing, we knocked down RelA expression by siRNA (Supplementary Fig. S6A) and analysed the expression levels of miR-23b cluster and miR-125a-5p by qPCR. Targeted knockdown of RelA induced miR-23b cluster and miR-125a-5p upregulation (Fig. 5f). Computational scanning predicted multiple NF-kB p65 binding sites in the promoter of miR-23b cluster and miR-125a-5p. To ascertain the binding activity of RelA to these sites causing a transcriptional block, miR-23b cluster and miR-125a-5p promoter sequence containing NF-kBp 65 binding sites were cloned into a promoterless reporter vector and co-transfected with a plasmid expressing a dominant active NF-kB p65 repressor, IkB-α (IkB-α-DA). A significant upregulation of the reporter gene activity was observed after NF-kB p65 inhibition compared to controls (*p* < 0.001, Fig. 5g). Because also total RelA was upregulated by PDGFR-α or PDGFR-β overexpression (Fig. 5e) we asked whether miR-23b cluster or miR-125a-5p could be involved in NF-kB p65 regulation. Using public available algorithms, we found that IKKβ (a NF-kB activator) and RelA were predicted to be miR-24 targets (Supplementary Fig. S6B). Overexpression of miR-24 in A549 cells markedly reduced IKKβ and NF-kB p65 phosphorylation and protein levels (Fig. 5h,i). In addition, luciferase reporter activity of IKKβ-3′UTR was significantly reduced after miR-24 transfection and it was rescued by the deletion of miR-24 binding site. The reporter gene activity of RelA 3′UTR presented only a marginal downregulation after miR-24 enforced expression (Fig. 5h,i), denoting that miR-24 silences IKKβ directly and RelA indirectly. Therefore, NF-kB p65 negatively regulates the expression of miR-23b cluster and miR-125a-5p through a direct binding to their promoter regions. In turn, miR-24 silences IKKβ and RelA establishing a negative feedback loop.

### MiR-23b cluster and miR-125a-5p as potential therapeutic tools

**Figure 6:**
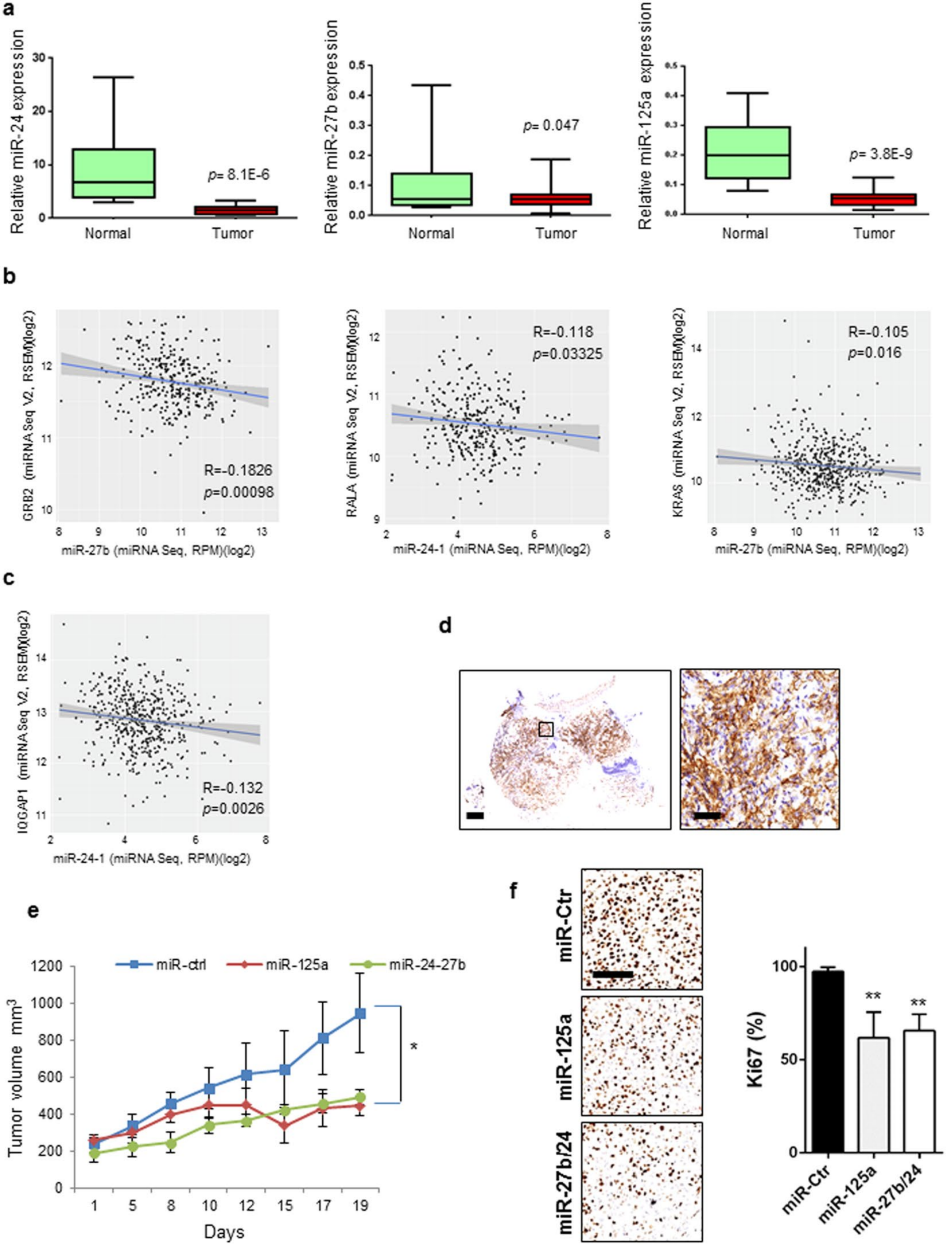
Effect of miR-23b cluster and miR-125a-5p on lung tumorigenesis *in vivo*. (**a**) Box plots showing relative expression of mature miR-24, miR-27b and miR-125a-5p expression in 24 normal lung or 24 lung tumour tissues. (**b,c**) Scatter plots showing inverse correlation between miRNA and respective target gene in 323 (GRB2/miR-27b and miR-24-1/RALA) or 513 (miR-27b/KRAS and miR-24-1/IQGAP1) patients with NSCLC from the Cancer Genome Atlas (TCGA) data set LUAD (lung adenocarcinoma). (**d**) Representative images of CDX sections showing strong expression of PDGFR-β in cancer cells (Original magnifications: left panel 2x, right panel 8x of left panel insert; scale bars 250 µm). (**e**) Tumour volume of cohorts of CDX mice treated with a control miR (Ctr), miR-125a-5p or miR-27b/24. (**f**) Representative images of Ki67 expression in CDX tumors treated with control miR versus miR-125a-5p or miR-27b/24. (Original magnifications: 20x; scale bar 100 µm). Average Ki67 evaluation in the three groups (Ctr *vs* miR-125a: p < 0.001; Ctr *vs* miR-27b/24: p < 0.001). Error bars indicate mean ± SD and p values were calculated by two-tailed Student t test (*p < 0.05, **p < 0.001). *r* = Pearson correlation coefficient.

Next, we analysed the expression of the components of miR-23b cluster and miR-125a-5p mature microRNAs in a cohort of lung tumors compared to the normal counterparts. MiR-24, miR-27b and miR-125a-5p were significantly downregulated in the tumour compared to normal lung samples (Fig. 6a). A statistically significant inverse correlation between microRNA and target gene was also found in the same tumour samples (*p* < 0.05, Supplementary Fig. S7A) and in a larger dataset (LUAD) from the TCGA (http://gdac.broadinstitute.org) (Fig. 6b and c). To test the therapeutic potential of miR-23b cluster and miR-125a-5p *in vivo* we employed a CDX (Circulating tumor cell patient Derived eXplant) NSCLC model derived from a NSCLC patient with advanced metastatic disease^24^. CDX tumours were genetically similar to the donor patient’s primary tumour which was EGFR, KRAS and ALK wild type and unresponsive to cisplatin and pemetrexed^24^. We analysed PDGFR-α and PDGFR-β expression in the CDX and found high expression of PDGFR-β (Fig. 6d) but not PDGFR-α (data not shown). Members of miR-23b cluster and miR-125a-5p were significantly downregulated in the CDX compared to normal lung (Supplementary Fig. S7B). Intratumoral injections of miR-24 and miR-27b or miR-125a-5p, without any drug treatment, significantly reduced tumor growth as assessed also by a decrease in proliferation (Ki67) and tumor weight (*p* < 0.05, Fig. 6e and f; Supplementary Fig. S7C), suggesting that these microRNAs may have therapeutic potential in NSCLC.

## Discussion

**Figure 7:**
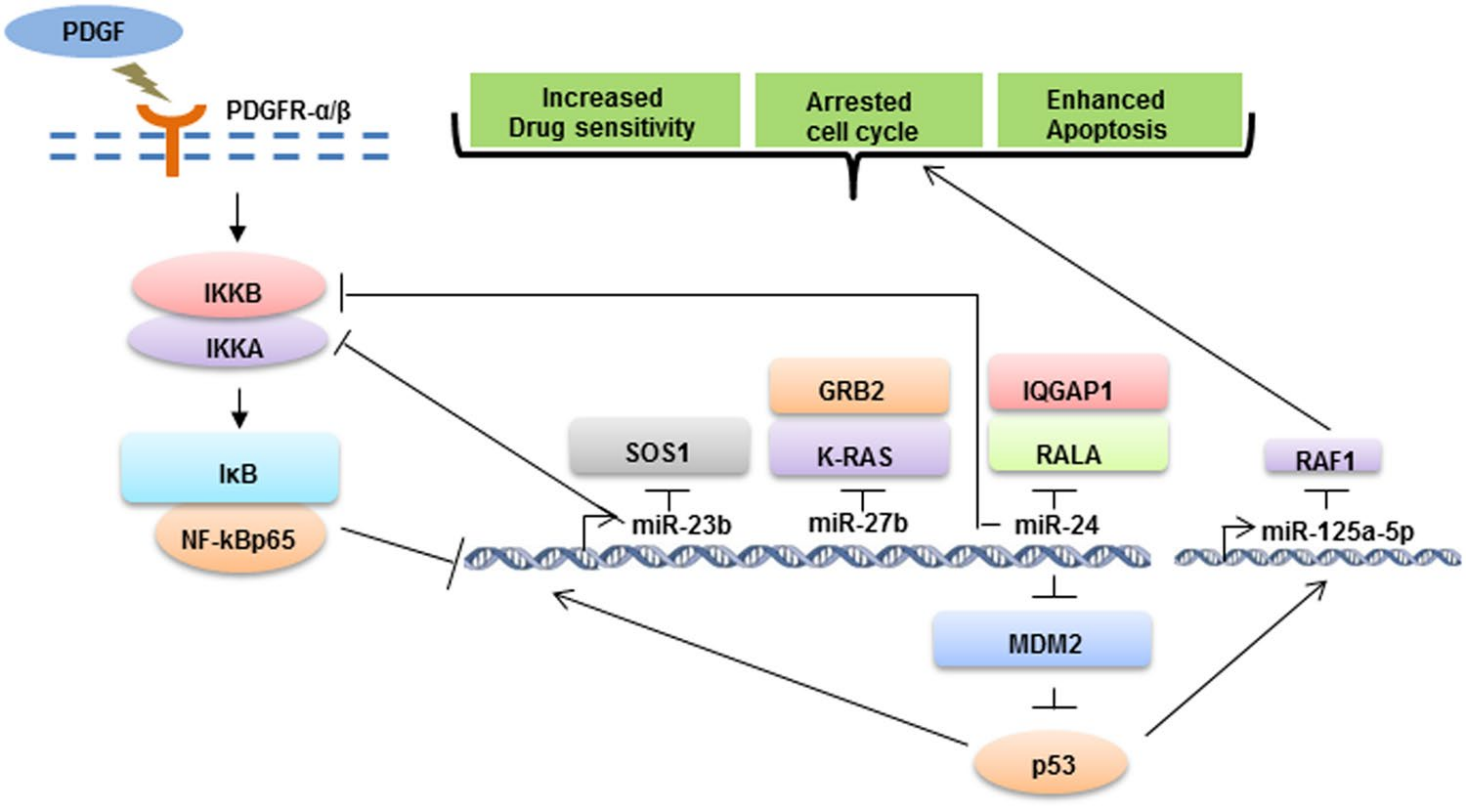
Model depicting the regulatory network controlling miR-23b cluster and miR-125a-5p. PDGFR-α and PDGFR-β downregulate miR-23b cluster and miR-125a-5p via NF-kB p65. MiR-23b cluster and miR-125a-5p are transcriptionally activated by p53 and silence several important oncogenes involved in the KRAS and NF-kB pathways.

PDGFRs are expressed frequently by tumor-associated stromal cells and by cancer cells in a subset of lung tumors^25^ and their overexpression or mutations are markers of worse prognosis^6^. PDGFRs exert proliferative effects through KRAS, the most frequent mutated oncogene in NSCLC^26^. In this study, we have shown that PDGFRs regulate the KRAS pathway through miR-23b cluster and miR-125a-5p, which directly silence KRAS signaling components such as SOS1, GRB2, IQGAP1, RALA, RAF1 and KRAS itself. Importantly, a significant inverse correlation between these microRNAs and respective target gene was also found *in vivo* in a large number of adenocarcinoma samples. Given the fact that “locked in” KRAS activation drives tumour progression in NSCLC, several targeting approaches such as blocking the activity of effector molecules in the KRAS pathway, enhancing KRAS-GTPase interactions^27^ and directly knocking down mutant KRAS by siRNA^28^, have been employed to inhibit its oncogenic activity. Despite these extensive efforts, KRAS remains largely undruggable for cancer therapy for the reasons including, but not limited to, pharmacological and biological constraints associated with this pathway^29^. One approach has been based on developing drugs or combinations of agents that work downstream of activated KRAS, for example blocking the ERK/MEK pathway. However, treatment with a MEK inhibitor, selumetinib, showed little clinical efficacy in a phase II clinical trial^30^. PI3K pathway is another promising RAS downstream target but combination of AKT and ERKs inhibitor only partially restored sensitivity to gefitinib in NSCLC cell lines^31^. Recently, it was suggested that the scaffold protein IQGAP1 may be another molecule downstream of RAS that can also be targeted^32^ indicating that several oncogenic pathways should be blocked simultaneously to stop tumor growth. However, very often drug combinations might induce the appearance of side effects in patients, which worsen quality of life or lead to the interruption of the treatment^30^. In this study, we proved that miR-23b cluster and miR-125a-5p can switch off simultaneously AKT, ERKs and KRAS signalings by silencing many of the components activated by KRAS in both KRAS mutant and wild type cells. Indeed, miR-23b cluster and miR-125a-5p inactivated the downstream pathways modulated by KRAS including RAF1/ERKs, AKT and RALGefs. Previous studies have correlated constitutive activation of these pathways to drug resistance^33,34^. In line with this notion, enforced expression of miR-23b cluster and miR-125a-5p significantly reduced proliferation and this effect was increased by treatment with cisplatin, pemetrexed, the combination cisplatin plus pemetrexed or TRAIL treatment in NSCLC cell lines, whereas miR-23b cluster and not miR-125a-5p increased cisplatin-mediated apoptosis. Oncogenic KRAS signaling promotes cell cycle progression^35^. Enforced expression of miR-23b did not show any significant change in cell cycle progression but miR-24 led to a significant enrichment of cells at G2/M phase whereas miR-27b and miR-125a-5p blocked the cells in G1 phase. p21 is considered as a negative regulator of the cell cycle and plays an essential role in G2/M phase arrest^20^. We found that p21 levels increased after miR-24 forced expression. It is known that the expression of p21 is negatively regulated by MDM2 and AKT^36^. Because, miR-24 silences AKT and MDM2 (Figs 2b and c, 5d), we can speculate that miR-24-dependent G2/M arrest may be mediated via MDM2 and AKT. Inhibition of KRAS and RAF1 has previously shown to cause G1/S arrest and indeed we found a G1/S phase arrest after miR-27b and miR-125a-5p enforced expression as a consequence of KRAS and RAF-1 downregulation^37^. Furthermore, we have demonstrated that miR-23b cluster and miR-125a-5p are p53 responsive miRNAs. Inhibition of MDM2 by nutlin 3 increased the expression of miR-23b cluster and miR-125a-5p. On the other hand, ectopic expression of miR-24 silenced the endogenous levels of MDM2, a known negative modulator of p53, enhancing p53 phosphorylation at serine 15 and establishing a positive feedback loop. Importantly, PDGFR-β silencing in H1703 cells increased miR-23b cluster and miR-125a-5p expression levels; therefore PDGFRs modulate the expression of these microRNAs in physiologic conditions. However, H1703 cells are p53 mutant cell lines, suggesting that the activation of miR-23b cluster and miR-125a-5p in the absence of p53 is regulated by other transcription factors. KRAS mutated tumors activate NF-κB p65 pathway to produce anti-apoptotic signals^38^ and inhibition of NF-kB p65 activation sensitized NSCLC to chemotherapy-induced apoptosis^39^. The intimate link between NF-kB p65 and PDGFR signaling has previously been documented^23^. We demonstrated that PDGFR-α or PDGFR-β induced downregulation of miR-23b cluster and miR-125a-5p through NF-kB p65 activation. On the other side, miR-24 blocked NF-kB p65 signaling by targeting directly IKKβ and indirectly RelA, evidencing the existence of a negative regulatory loop. Interestingly, another central regulator of NF-kB p65 activation, IKKα, has previously been reported as target of miR-23b, although in a different system^40^. Based on these data, we propose that two inter-dependent regulatory circuits are employed in the regulation of miR-23b cluster and miR-125a-5p expression (Fig. 7). A positive feedback mechanism mediated by p53-miR-24-MDM2 activates miR-23b cluster and miR-125a-5p expression, whereas, a NF-kB p65-mediated negative regulatory loop blocks the transcriptional activation of both miR-23b cluster and miR-125a-5p. Importantly, previous studies have reported that AP-1 and c-Myc are also able to suppress the expression of miR-23b cluster, confirming they act as “bona fide” tumor suppressor microRNAs^41,42^. EMT is a multistep process associated with metastasis and drug resistance. We demonstrated that stable expression of miR-125a-5p and not of miR-23b cluster was able to induce a reversion of A549 to an epithelial phenotype, as assessed by the change in cell morphology, decrease of mesenchymal markers and increase of E-cadherin expression. Finally, we have shown the therapeutic potential of miR-23b cluster and miR-125a-5p using a CDX, generated by injecting circulating mesenchymal tumor cells obtained from the blood of a NSCLC patient into athymic immunocompromised mice that faithfully recapitulates patient response to standard chemotherapy^24^. Intratumoral injection of miR-24 and miR-27b or miR-125a-5p significantly reduced the growth of this aggressive tumor without evidence of toxicity. In summary, here we provide strong evidence that miR-23b cluster and miR-125a-5p act as “bona fide” tumor suppressor microRNAs by silencing directly or indirectly several oncogenic redundant pathways and main effectors of tumorigenesis in NSCLC. Our data prove that miR-23b cluster and miR-125a-5p can efficiently and specifically switch off simultaneously PDGFRs, KRAS and NF-kB p65 downstream signaling and could be exploited as potential therapeutic tools in NSCLC.

## Methods

### Cell culture and reagents

All cell lines used in this study were purchased from ATCC or identification was performed on established lines using PowerPlex®21 system (Promega). Lines were tested for Mycoplasma every three months using the Venor®*GeM* Classic Mycoplasma detection Kit (Cambio Ltd). A549, H1703 and H1299 cell lines were cultured in RPMI medium (Gibco) supplemented with 10% fetal bovine serum (FBS) and 2 mM L-glutamine and 100 U/mL penicillin-streptomycin. HEK293 cells were grown in DMEM supplemented with 10% FBS and 100 U/ml penicillin-streptomycin. Cell cultures were maintained at 37 °C in a humidified chamber at 5% CO_2_. Unless otherwise mentioned, all chemicals and drugs were purchased from Sigma.

### *In silico* analysis

*In silico* prediction of miRNA targets was performed with TargetScan, PicTar, miRanda and PITA algorithms using the HUGO gene symbol as common identifier. Level 3 TCGA datasets of miRNA expression (miRNA Seq, RPM) (log2) or mRNA expression (mRNA Seq V2, RSEM) (log2) for lung adenocarcinoma (LUAD) samples were downloaded from The Broad Institute Genome Data Analysis Center (GDAC) [doi:10.7908/C11G0KM9]. Using custom scripts 325 (N0) or 513 samples were extracted from TCGA to analyse inverse correlation between microRNA and target gene. For normally distributed data, significance was obtained using ttest otherwise Mann–Whitney test was used to assess significance.

### Clinical samples

24 primary NSCLC and 24 normal lung tissue samples were collected from the Department of Pathology, The Ohio State University (OSU). The experimental protocol (#2005C0014) was approved by the Ohio State University Review Board and carried out in accordance with the OSU guidelines and regulations. Informed consent was obtained from all patients before the study. Tissue samples were fresh-frozen in liquid nitrogen after surgery and kept at −80 °C. Frozen tissue samples were homogenized using the Tissue Ruptor (Qiagen) before RNA extraction. Total RNA was extracted using Trizol (Invitrogen) in accordance with manufacturer’s instructions.

### *In vivo* studies

The CDX employed in this study was initially generated using circulating tumor cells (CTCs) from a 48-year-old male recruited through an ethically approved translational lung cancer research programme as previously described^24^. The patient underwent chemotherapy with cisplatin and pemetrexed but discontinued after one cycle with deterioration of his general condition and died 2 months after initial diagnosis. Blood was drawn before administration of chemotherapy for CTC enrichment before implantation into immunocompromised mice^24^. The CDX was passaged with maintained tumor growth dynamics and at sacrifice CDX cells were dissociated and 1 × 10^5^ implanted s.c. for the experiments described here. When the tumours reached 200 mm^3^, mice (n = 4) were randomized by sequential assignment to miR-Ctr, miR-27b/24-1 and miR-125a-5p treatment groups and injected with *in vivo* jetPEI (Polyplus) complexed with 100 µg of each microRNA four times in three weeks. Tumor volume was determined by measuring the length and the width of the tumor mass and calculating the volume [volume = (width)^2^ (length)/2]. At the end of the experiment, mice were euthanized and tumours were harvested for analysis. All procedures involving animals were approved by CRUK Manchester Institute’s Animal Welfare and Ethical Review body in accordance with the ARRIVE guidelines^43^ and the Committee of the National Cancer Research Institute guidelines^44^ and conducted under project license number 70/8252 (C.D.).

### Luciferase reporter assay

3′-UTRs of RALA, KRAS, SOS1, IQGAP1, GRB2, RAF1, AKT2, ERK2, MDM2, IKKβ and RelA were PCR amplified from human genomic DNA (Promega) using primers reported in Supplementary Table 1 and inserted into the pGL3 control vector (Promega). Deletions of miRNA binding sites were performed using a Quick-Change Mutagenesis Kit (Stratagene). MiR-23b cluster (genomic locations: 95084241–95085020 (780 bp)) or miR-125a-5p promoter regions (51690785–51691283 (499 bp)) containing p53 binding sites were cloned into a promoterless reporter vector (PGL3 basic). MiR-23b cluster (genomic locations: site1–95081977 to 95082482 (506 bp), site2–95082802 to 95083131 (330 bp) and site3–95084396 to 95084985 (590 bp)) or miR-125a promoter regions (genomic locations: site1–51686289 to 51686703 (415 bp), site2–51690234 to51690825 (592 bp) and site3–51692155 to 51692992) containing NF-kBp65 binding sites were cloned into a promoterless pGL3 basic reporter vector (Promega) and co-transfected with a plasmid expressing a dominant active NF-kBp65 repressor, IkBα in A549 cells. Luciferase activity was tested with Dual Luciferase Assay (Promega) according to the manufacturer’s instructions.

### Generation of stable cell lines

For the generation of stable cell lines, miR-23b cluster and miR-125a-5p sequences were PCR amplified (primers are listed in Supplementary Table 1) and cloned into pmR-ZsGreen1 vector (Clontech). A549 cells were transfected with 1 µg of each plasmid and cells overexpressing miR-23b cluster or miR-125a were selected in 1 µM of G418.

### RNA isolation and reverse transcription quantitative PCR (RT-qPCR)

Total RNA was extracted using TRIzol solution (Ambion), according to the manufacturer’s instructions. For quantification of miRNA levels, 200 ng of total RNA were used for reverse transcription (RT), using the TaqMan MicroRNA Reverse Transcription Kit (Applied Biosystem) and real time PCR was performed using the TaqMan MicroRNA assay kit (Applied biosystem) as described previously^45^. Small Nucleolar RNA U44 or U48 were used to normalize the data. qPCR primers are listed in Supplementary Table 1.

### Transient transfection

Cells were transfected with 100 nM of miRNA or siRNA or 1 μg of plasmids using Lipofectamine 2000 reagent according to manufacturer’s instructions. Assays were performed either 48 h or 72 h post transfection. PDGFR-α and PDGFR-β expression vectors and siRNAs were purchased from Dharmacon.

### Immunoblotting

Cells were lysed in lysis buffer (150 mM NaCl, 30 mM TRIS, 0.1% Triton X-100, 10% glycerol and 1X protease inhibitor cocktail). Protein concentration was quantified using the BCA method (Pierce). 50 μg of total protein was resolved either on 4–12% NuPAGE^®^ Bis-Tris or 3–8% Tris-Acetate mini-gels (Invitrogen) and transferred to PVDF membrane. Membranes were blocked for 1 h at room temperature (RT) either with 5% non-fat dry milk or 5% Bovine serum albumin (BSA) in Tris-buffered saline containing 0.01% Tween 20 (TBS-T) and incubated overnight at 4 °C with primary antibodies. Chemiluminescence was detected using Novex® ECL substrate reagent and High performance film (GE Healthcare). β-actin, α-tubulin or GAPDH were used as loading controls. Antibodies for PDGFR-α (#5241), PDGFR-β (-#3169), GRB2 (#3972), pERKs (#9101), RAS (#8955), pAKT (#4060), totAKT (#2966), AKT2(#5239), pp53 (Ser15) (#9284), pRelA (#3033), totRelA (#6956), IKKβ (#8943), GAPDH (#3683) were purchased from Cell Signaling Technology. SOS1 (ab140621), IQGAP1 (ab86064), ERK2 (ab124362), p21 (ab109520), CDK1 (ab19), p53 (ab179477) antibodies were obtained from Abcam. MDM2 antibody was purchased from Calbiochem (OP115). RALA (sc-374462), Raf-1(sc-227) and β-actin (SC-47778) antibodies were purchased from Santa Cruz Biotechnology.

### Chromatin-Immunoprecipitation (ChIP) assay

A549 cells (1 × 10^6^ cells) were cross-linked with 1% formaldehyde at RT for 10 min with gentle rocking. Cross-linking was quenched by adding glycine to a final concentration of 0.125 M for 5 min at room temperature. Cells were washed twice in ice cold PBS with protease inhibitors and then lysed with buffer containing (1% SDS, 10 mM EDTA, 150 mM NaCl, 50 mM TRIS-HCl (pH 8.1) and 1X protease inhibitor cocktail. Lysates were subjected to sonication (Bioruptor; Diagenode) to yield chromatin fragments of average size ~500–600 bp. Protein A beads (Invitrogen) were incubated overnight at 4 °C either with IgG control (Abcam) or rabbit polyclonal antibody for human p53 (Abcam), and then washed extensively with IP dilution buffer (1% Triton X-100, 2 mM EDTA, 150 mM NaCl and 20 mM TRIS pH 8.1 and 1X protease inhibitor cocktail). Sheared chromatin was added to the antibody/IgG control bound beads and incubated at 4 °C for 2 h with gentle rotation. Beads were washed sequentially three times at 4 °C with washing buffer I (0.1% SDS, 1% Triton X-100, 2 mM EDTA, 150 mM NaCl and 20 mM TRIS pH 8.1), washing buffer II (0.1% SDS, 1% Triton X-100, 2 mM EDTA, 500 mM NaCl and 20 mM TRIS pH 8.1) and washing buffer III (0.25 M LiCl, 1% NP40, 1% deoxycholate, 1 mM EDTA and 10 mM TRIS pH 8.1) and finally three times with Tris-EDTA. DNA was eluted and purified using IPure kit (Diagenode). DNA binding by p53 was analysed by qPCR using SYBR-green and primers, listed in Supplementary Table 1, covering regions of p53 binding sites on miR23b cluster or miR125a-5p promoter. Results were expressed as fold change above background and normalized to control (IgG) levels.

### NanoString

The NanoString nCounter Human miRNA Expression Assay Kit (http://www.nanostring.com/) has been employed to identify altered miRNAs expression after PDGFR-α and PDGFR-β overexpression. 100ng of total RNA was used for nCounter miRNA sample preparation according to manufacturer’s instructions (NanoString Technologies). Briefly, unique DNA tags were ligated to the 3′ end of each mature miRNA. After ligation, samples were cleaned to remove excess tags and hybridized to a panel of miRNA:tag-specific nCounter capture and barcoded reporter probes. Fluorescence was collected on the nCounter Digital Analyzer (NanoString Technologies) from individual fluorescent barcodes and quantified target RNA molecules present in each sample. For each assay, a high density scan (600 fields of view) was performed. Raw data was normalized to top-100 expressed miRNA as implemented by the NanoString nSolver software. P-values were calculated using the LIMMA package (Linear Models for Microarray Data) from the Bioconductor R project^46^. Normalized miRNA expression data was visualized by the heatmap generated using hierarchical clustering module of GenePattern software^47^. Pearson correlation was used as a distance metric and pairwise complete-linkage as a clustering method.

### Cell viability assay

5 × 10^3^ A549 or H1299 cells were cultured in 96-well plate and transfected either with negative control or with miR-23b, miR-24, miR-27b or miR125a-5p mimics. 48 h after transfection cells were treated either with DMSO and 10 μM cisplatin (Sigma), 2uM pemetrexed (Selleck Chemicals) or 100 ng/mL of TRAIL for 24 h. Cell viability was determined using CellTiter 96^®^ Aqueous One Solution Cell Proliferation Assay (Promega) according to manufacturer’s instructions. Metabolically active cells were detected by adding 20 μl of MTS reagent to each well. Absorbance at 490 nm was analyzed in a Multilabel Counter (SpectraMax M5).

### Annexin-V FITC Apoptosis assay

Cells were transfected either with a microRNA control or with miR-23b, miR-24, miR-27b or miR-125a-5p mimics. 48 h after transfection cells were treated with 10 μM of cisplatin (Sigma) for 24 h and samples were analysed in triplicates on FACS-Calibur (BD biosciences) using TACS^®^ Annexin V kit following the manufacturer’s instructions.

### Cell cycle analysis

MiR-23b, miR-24, miR-27b and miR125a-5p or negative control mimics were transfected into A549 cells. 72 h after transfection cells were trypsinized, washed with 1X ice-cold PBS, stained with propidium iodide (PI) staining buffer (PBS, 0.1% Triton X-100, 0.5 mg/ml propidium iodide) and incubated at 37 °C for 1 h. Results were obtained using 4 colour Calibur device (BD) and analysed with Flowjo software (Flowjo Enterprise).

### Invasion assay

Invasion assay was performed using InnoCyte invasion kit as per manufacturer instructions (Millipore). Briefly, A549 cells were transfected either with negative control or with miR-23b, miR-24, miR-27b or miR125a-5p mimics for 48 h. Cells were trypsinized and resuspended in serum-free medium and seeded in the upper chambers and media containing serum was added to the lower chamber as chemoattractant. Invasion assays were performed as described previously^48^.

### Colony assay

A549 cells stably expressing an empty vector (eV), miR-23b cluster or miR-125a-5p were seeded into 10 cm dishes at a density of 4000 cells/dish. Eight days later cells were fixed with ice-cold methanol for 5 min at room temperature and stained with crystal violet for 20 min. Excess stain was washed with tap water and images of the dishes acquired using GelCount (Oxford Optronix).

### Statistical analysis

Two-tailed student’s t test or Mann–Whitney test were used to determine significance. A P value of ≤0.05 was defined as statistically significant. The Pearson’s correlation was calculated using the GraphPad Prism package (GraphPad Software Inc) and R software package for statistical analysis Version 3.1.0.

### Data availability

All data generated or analysed during this study are included in this article (and its Supplementary Information files).

## Electronic supplementary material

Supplementary Information
