# Supplementary material for: PDGFR-modulated miR-23b cluster and miR-125a-5p suppress lung tumorigenesis by targeting multiple components of KRAS and NF-kB pathways
Source: Sci Rep. 2017 Nov 13;7:15441. doi: 10.1038/s41598-017-14843-6 (PMC5684387; doi:10.1038/s41598-017-14843-6)

Supplementary Information

PDGFR-modulated miR-23b cluster and miR-125a-5p suppress lung tumorigenesis by targeting multiple components of KRAS and NF-kB pathways.

Srivatsava Naidu, Lei Shi, Peter Magee, Justin D. Middleton, Alessandro Lagana', Sudhakar Sahoo, Hui Sun Leong, Melanie Galvin, Kristopher Frese, Caroline Dive, Vincenza Guzzardo, Matteo Fassan and Michela Garofalo

Supplementary Figures 1S-7S

Supplementary Table 1S

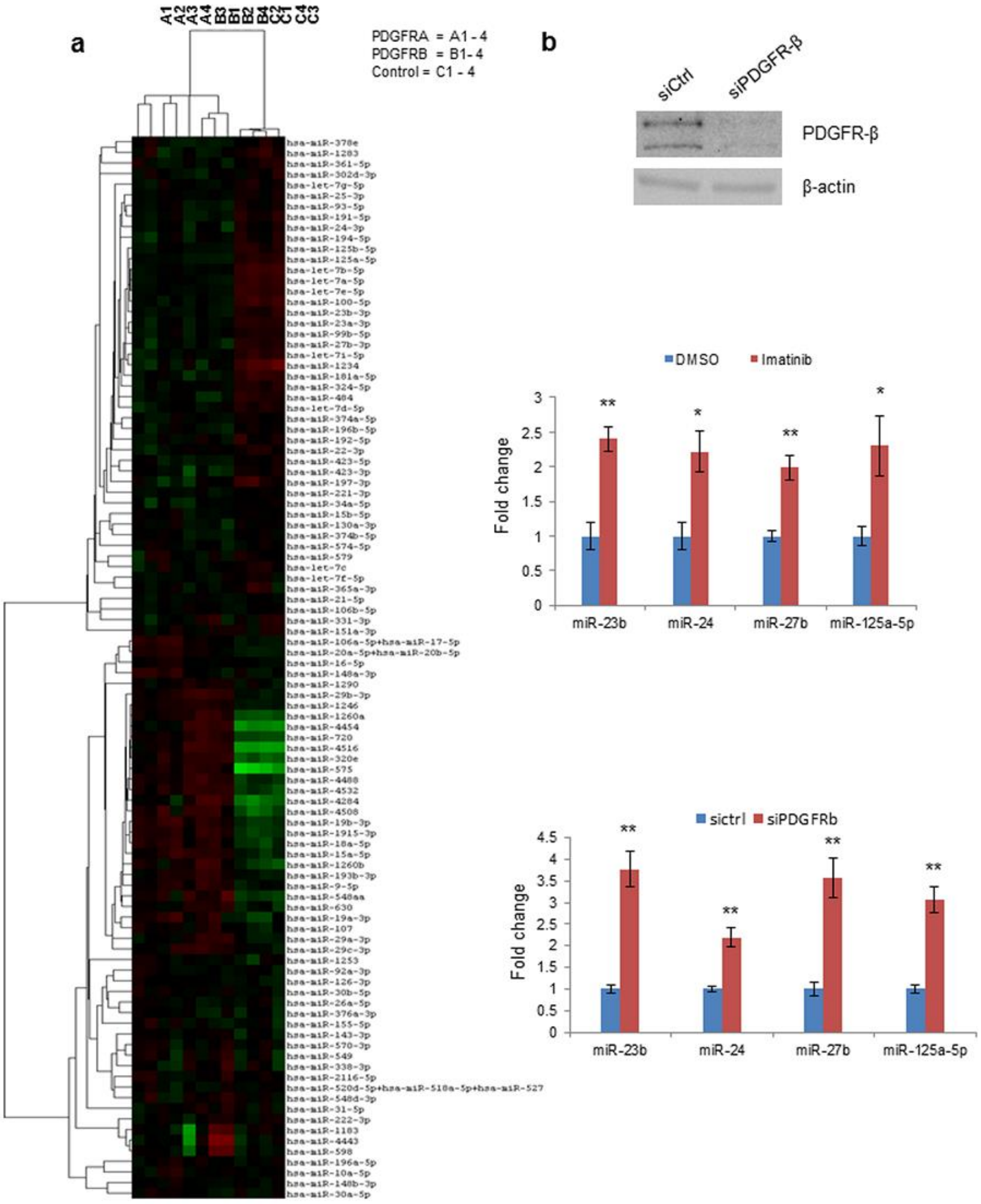

Supplementary Figure 1. MicroRNA expression profile after PDGFR-α or PDGFR-β overexpression. a) Heatmap showing unsupervised hierarchical clustering of miRNAs expression in A549 cells after overexpression of PDGFR-α or PDGFR-β or empty vector as control. P values were obtained by Anova ($p < 0.05$). **b)** MiR-23b cluster and miR-125a-5p are upregulated after imatinib treatment or PDGFR-β silencing in H1703 cells. Error bars indicate ± SD (n=3). *p<0.005, **p<0.001 by two-tailed Student's t test.

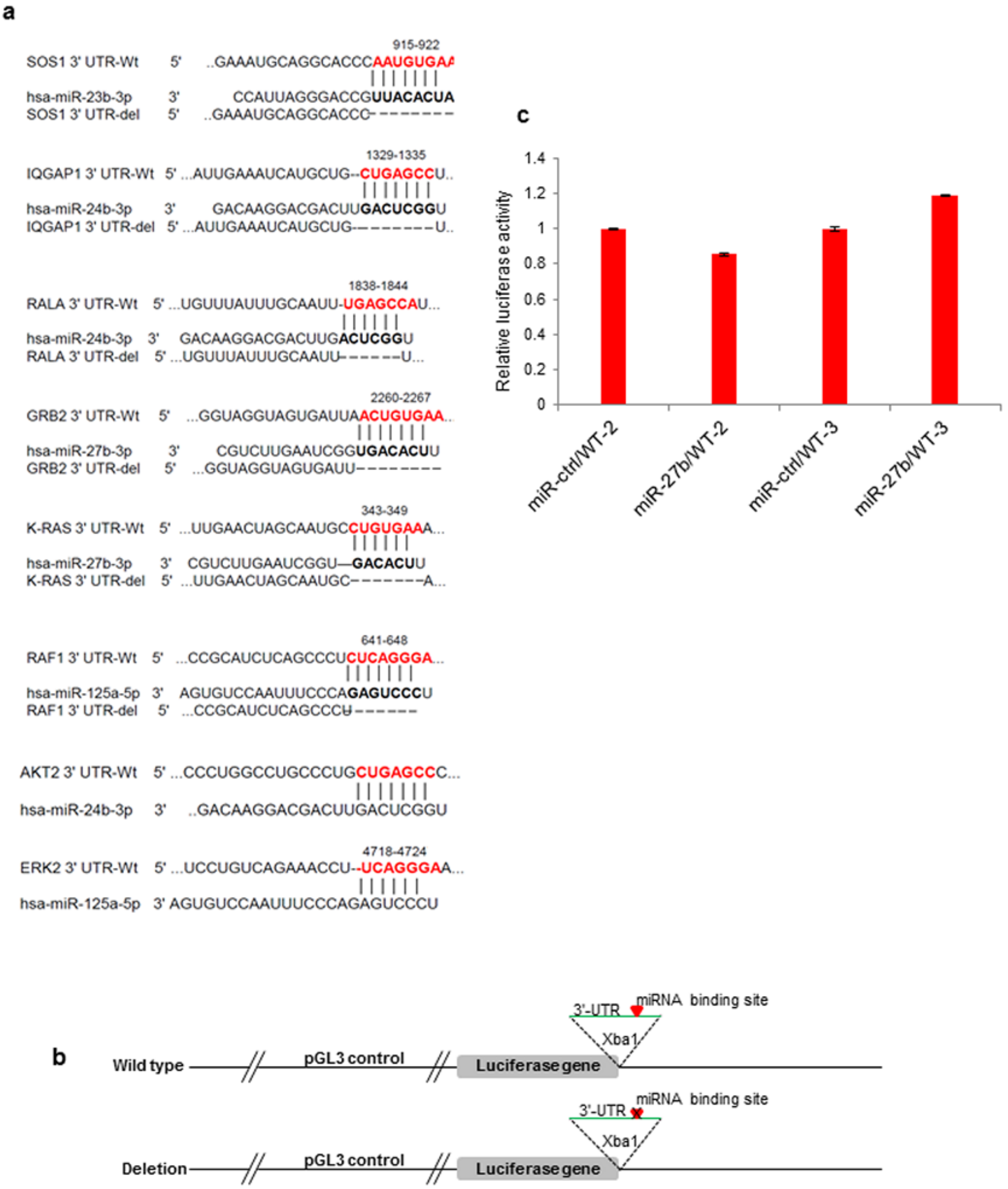

Supplementary Figure 2. MiRNA seed sequence in the 3' UTRs of target genes. a) In the figure the alignment of the seed regions of miRNAs with the respective target 3'UTR is shown. The sites of target mutagenesis are indicated in red. Deleted nucleotides are indicated as -. **b)** Schematic representation of luciferase reporter vectors containing miR-23b cluster and miR-125a-5p messenger RNA target 3'UTRs. **c)** Luciferase assay showing weak or no regulation for miR-27b in binding sites 2 and 3 of KRAS 3'UTR.

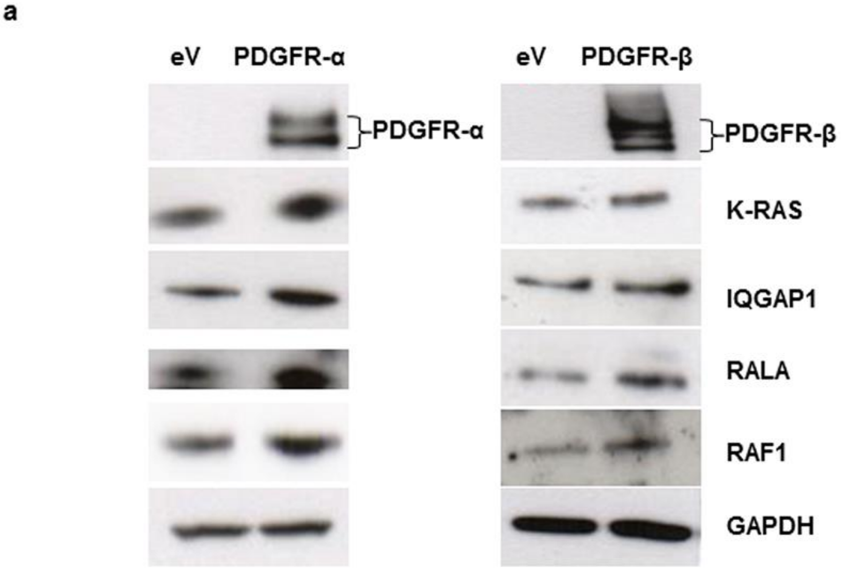

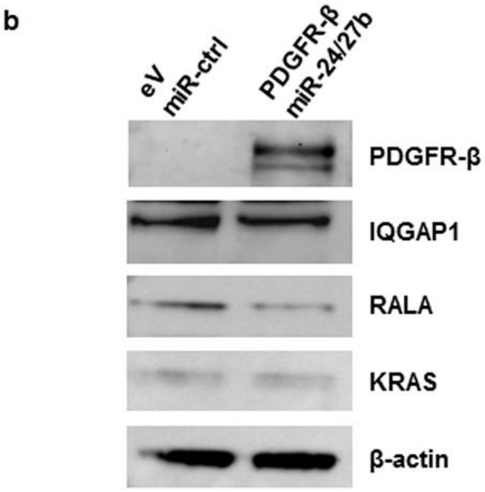

Supplementary Figure 3. Overexpression of PDGFR-α and PDGFR-β induces upregulation of miR-23b cluster and miR-125a-5p target genes. a) A549 cells were transfected with either an empty vector (eV), PDGFR-α or PDGFR-β for 48 h. Overexpression of PDGFR-α or PDGFR-β induced upregulation of KRAS, IQGAP1, RALA and RAF1 as a result of miR-23b cluster and miR-

125a-5p downregulation. **b)** Enforced expression of PDGFR- β and miR-24/27b in A549 cells rescued the effect on target genes upregulation.

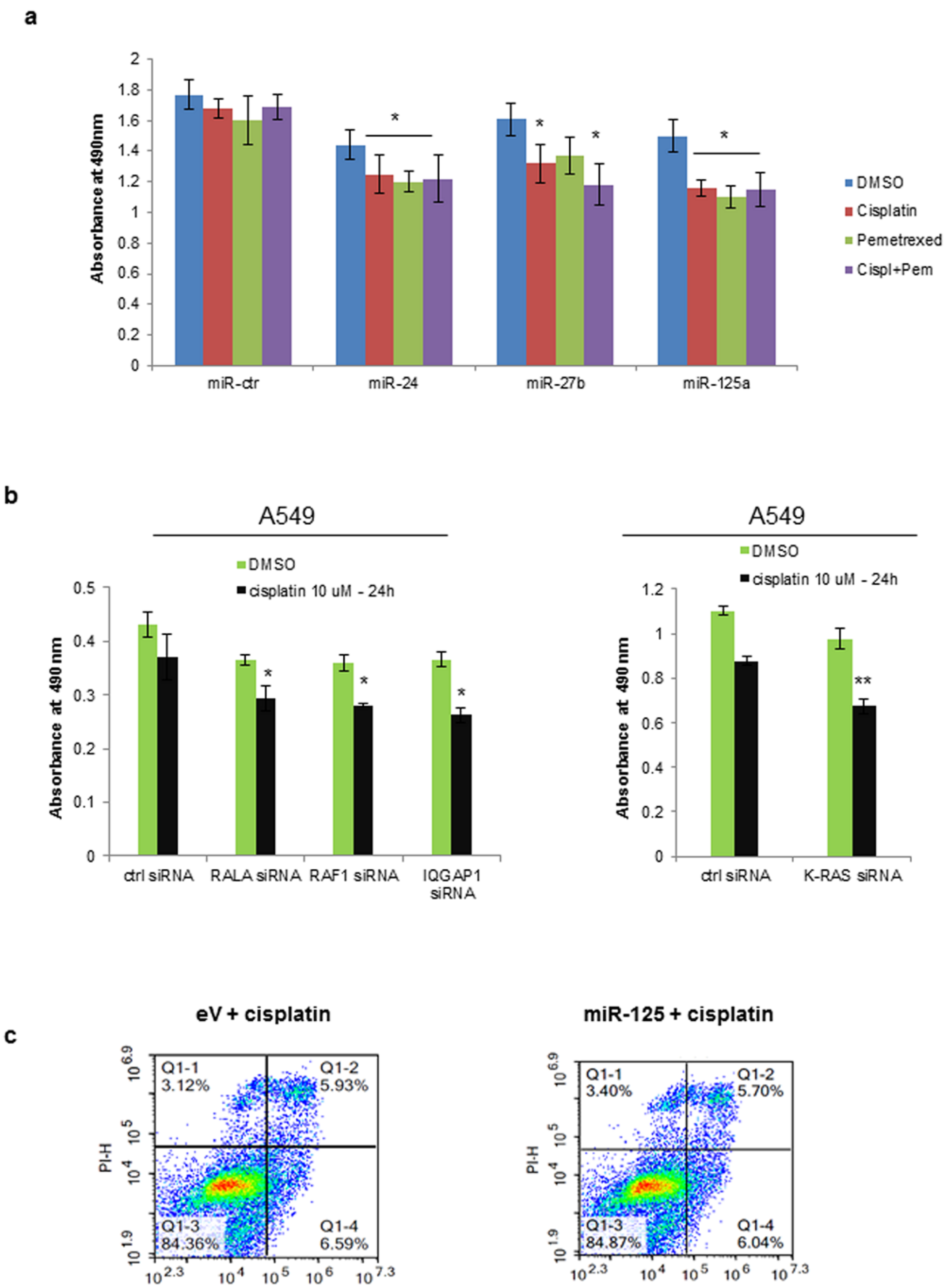

Supplementary Figure 4. KRAS, RALA, RAF1 and IQGAP1 silencing reduces cell proliferation

and increases drug response. a) Proliferation assay in A549 cells after miR-24, miR-27b or miR-125a-5p enforced expression and treatment with cisplatin, pemetrexed or the combination cisplatin/pemetrexed**. b)** A549 cells were transfected with RALA, RAF1, IQGAP1 and KRAS or control siRNA. 48h later, cells were treated with 10 µM of cisplatin for 24 h and viability was assayed using MTS assay. **c)** MiR-125a-5p upregulation has no effect on apoptosis. A549 cells stably expressing miR-125a-5p were treated with cisplatin and apoptosis was assessed by Annexin V staining. Experiments were performed at least three times in triplicates. Error bars indicate ± SD (n=3). **p*<0.005, ***p*<0.001 by two-tailed Student's t test.

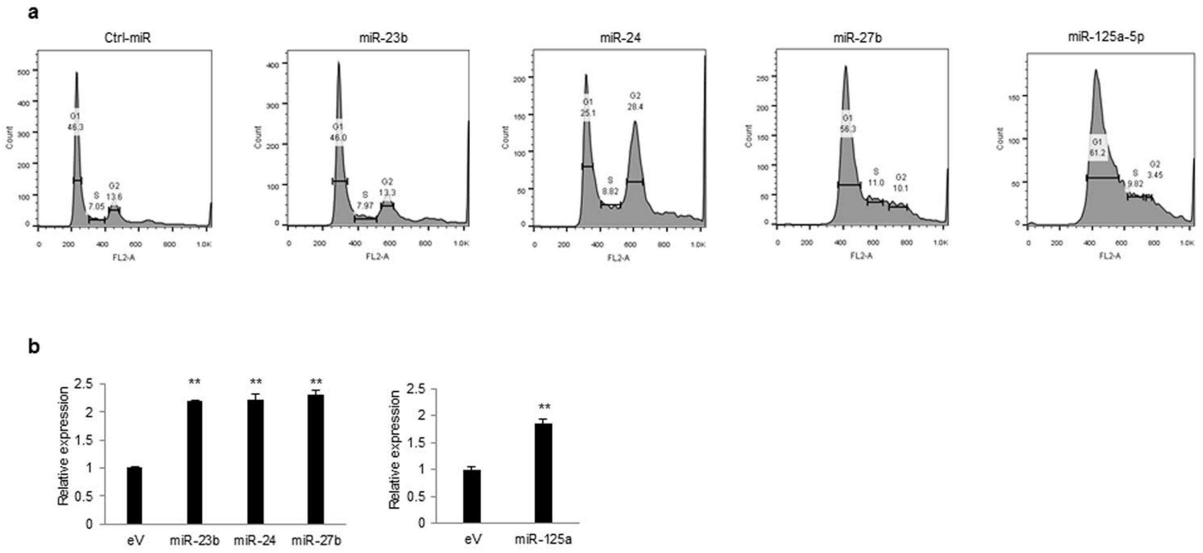

Supplementary Figure 5. Effect of miR-23b cluster and miR-125a-5p enforced expression on cell cycle progression. a) Cell cycle analysis of A549 cells transiently transfected with miR-23b, miR-24, miR-27b or miR-125a-5p. MiR-23b did not affect cell cycle progression, miR-24 blocked the cells in G2 phase whereas miR-27b and miR-125a arrested cell cycle in G1 phase. **b)** MiR-23b, miR-24, miR-27b and miR-125a-5p expression levels in A549 stable cells. Error bars indicate ± SD (n=3). ***p*<0.001 by two-tailed Student's t test.

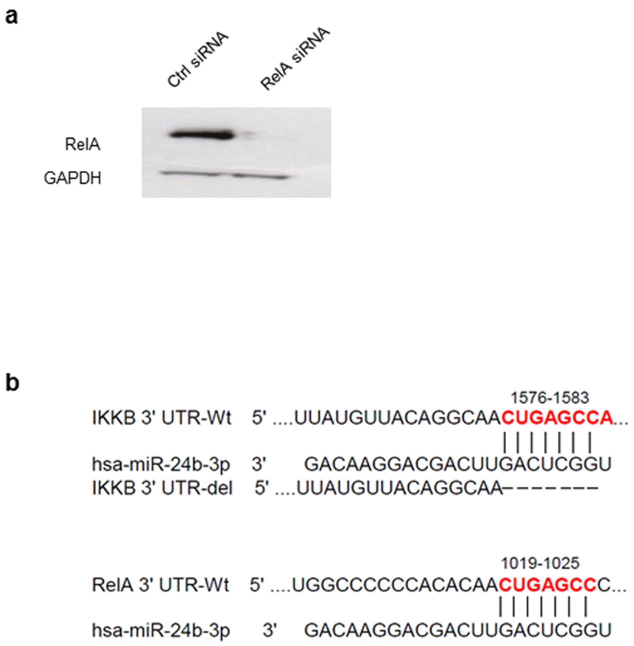

Supplementary Figure 6. MiR-24 seed sequence in the 3’ UTRs of IKKβ and RelA. a) RelA silencing by siRNA compared to cells transfected with a siRNA control. **b)** In the figure the alignment of the seed regions of miR-24 with IKKβ and RelA 3’UTRs is shown. The sites of target mutagenesis are indicated in red. Deleted nucleotides are indicated as -.

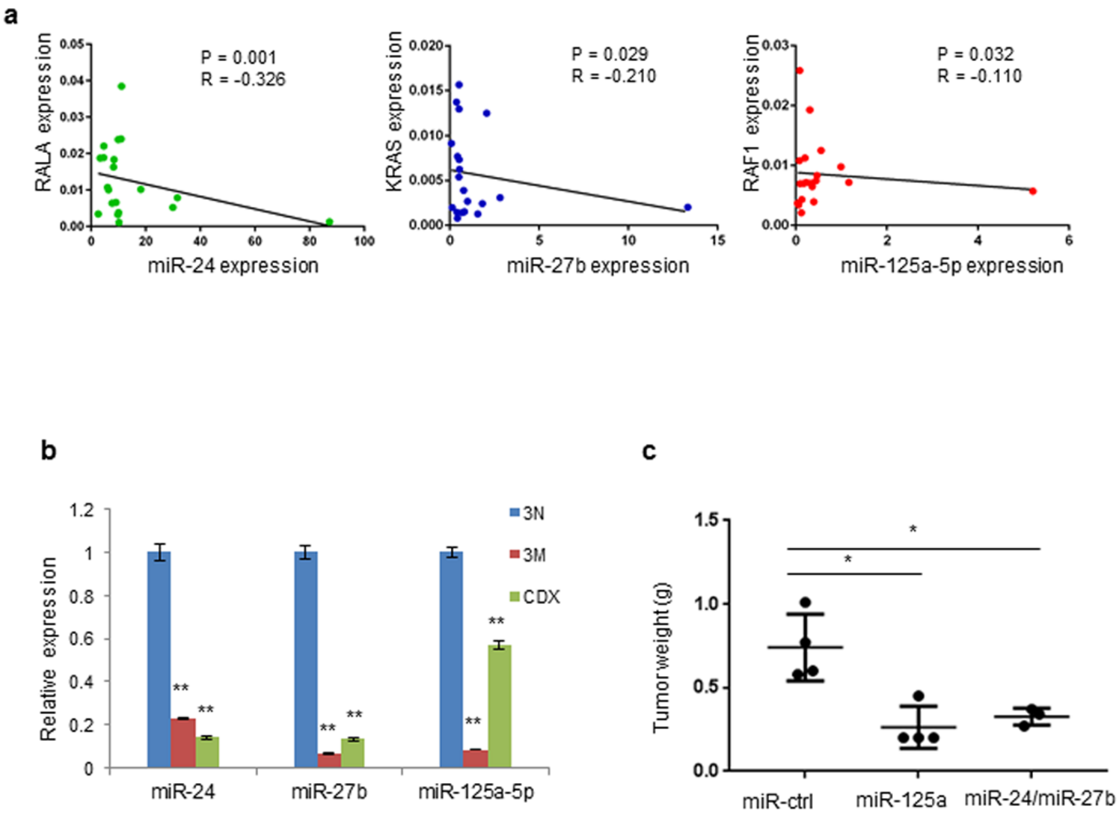

Supplementary Figure 7. A) Expression of miR-23b cluster and miR-125a-5p *in vivo*. a) Inverse relation between miR-24, miR-27b and miR-125a-5p and respective target genes in 24 lung tumours. **b)** qPCR showing downregulation of miR-23b cluster and miR-125a-5p in the CDX and tumor sample (3M) compared to normal lung (3N). **c)** Tumor weight for CDX mice treated with miR-ctrl, miR-125a-5p or miR-27b/24. Error bars indicate ± SD (n=3). **p* <0.005, ***p*<0.001 by two-tailed Student's t test. Ctrl=control. R= Pearson correlation coefficient.

Supplementary Table 1. Primers used in the study

	Sense (5'-3')	Antisense (5'-3')
Primers for 3'-UTR *Wt* constructs		
SOS1	GTGGTGGTGTTGCCTTCAAAA	AAACATGAGCGGTGTCAGTG
GRB2	ATCATGAAGCCTTGCTGAACT	TGAAGAATTCATTGTGTATTTATTA
IQGAP1	TTTTATGAGCCCCAGTTTTCTGGGCTTAG	GGCTGTAACCATGCACCTGAGTAAAGAGTCAAA
RALA	ACATCCAGCTTCTCATG	AAAAGGAAGTTTATTGG
K-RAS	TTCCTGCTCCATGCAGACTGTTAGC (BS1)	GGACATGATGCCTAGAAGAATCATC (BS1)
	ACA ACCGGT TGCTACGATTCCACTGAAAC (BS2)	TAT CTGCAG ACCCTACCTAAACAGTGTTCC (BS2)
	TAG ACCGGT TCCGAAAGTTTCCAATTCCAC (BS3)	TCT CTGCAG AAGGATACTTCTAACAAGCTGC (BS3)
RAF1		
AKT2	CATGGGATTTTGGAAATCAGCTTCTGGAGG	GGCTTCCTTGTATACACATGATGTGACTAG
ERK2	AAGAACCCTGTTCAGCTTGGG	AGGCCTGCCTATTTTATGACCA
MDM2	TGTTGTGAGGTGTCACTAAAATGC	ATTGCCTTATACAGAATCTGGACAACAG
IKKB	TTCTACTCTGTCTTAAATGAG	CTTTCACACATTTTCTCTTTATATC
RelA	CCCTGGTAAATGACAGTGGGA	TTGTCCTGTAACTGAACAGCCA
	GCTGAACCAGGGCATACCT	GAGAAGTCCATGTCCGCAAT
Primers for 3'-UTR *del* constructs		
SOS1_del	GGAAATGCAGGCACCCAAGGAGCACTTGCTGG	CCAGCAAGTGCTCCTTGGGTGCCTGCATTTCC
GRB2_del	CGGATAGCATTTGGTAGGTAGTGATTATAATAAATAC-ACAATGAATTCTTCATC	GATGAAGAATTCATTGTGTATTTATTATAATCAC-TACCTACCAAATGCTATCCG
IQGAP1	CCAGGAAAATATATTGAAATCATGCTGTCTATTTTCT-TTCTTTGATGTTTTGAT	ATCAAAACATCAAAGAAAGAAAATAGACAGCA--TGATTTCAATATATTTTCCTGG
RALA	AAAAAAGGAAGTTTATTGGTCTTTAAAAATTGCAAAT-AAACAAATCGGGTAGTG	CACTACCCGATTTGTTTATTTGCAATTTTTAAAG-ACCAATAAACTTCCTTTTTT
K-RAS_del	GTTTTGGTTTTTGAACTAGCAATGCAAAGAAACTGAA-TACCTAAGATTTC	GAAATCTTAGGTATTCAGTTTCTTTGCATTGCTA-GTTCAAAAACCAAAAC
RAF1	CAATCCGCATCTCAGCCAGCAGTCTTCCATCATG	CATGATGGAAGACTGCTGGCTGAGATGCGGATTG
AKT2	-	-
ERK2	-	-
MDM2	-	-
IKKB	CTGTGAGCTTATGTTACAGGCAACTGAAGAATTTTGA-CGAAGAAA	TTTCTTCGTCAAAATTCTTCAGTTGCCTGTAACA-TAAGCTCACAG
Primers for ChIP assays		
p53 at miR-23b-27b-24b promoter	ACACAGACTGTGCACTTACCTCACTGC	GATCCCTTCTCTCCAGGCAGAGATTCTAG
p53 at miR-125a-5p promoter	TTGAACAAGTCCTGACCCTTCTCTCTCCG	CTTCTCTCTCTCTCTTTTCTCCTTCCCTC
Primers for cloning of sequences expressing miR-23b cluster or miR-125a into pMR-ZS1 green expression vector		
miR-23b cluster	TTAGCAATTGTGTGTGTCGCA	AAGTCTCAGCTCACCTCGCC
miR-125a	CGGGCTCTTTTCTGTCCTTGT	GGCCATCGTGTGGGTCTCAA
Primers for cloning p53 binding sites in miR-23b cluster promoter or miR-125a promoter into pGL3 basic reporter vector		
miR-23b cluster	TGTGCTTATGAAAACCATTTCTG	TGCAGCTTTGGAACTGAAGA
miR-125a	CTTAGGTCCGGGGAATCTCT	CTCCTTCCCTCCCTCTTAGG
Primers for cloning NF-kB binding sites in miR-23b cluster promoter or miR-125a promoter into pGL3 basic reporter vector		
miR-23b cluster		
Site 1	TGTGAGTGTTTGCGGCATTG	ACTTGCTGGTCTAGAAATTGCA
Site 2	GTTAGCCAAGACTACCCGCA	CGGCCGCTTAAACTCCTGTA
Site 3	CTGAGCGTCTCGTAGCGAAA	GCCATGCGACACACACAATT
miR-125a		
Site 1	TTTTCCTGAGCCCCTGCTCTGAG	CTCACAAAGTATGTGATGGAGCT
Site 2	CAGGATCCAGGAGTCTGAGC	AGAGGGGAAGCCGAGAAT
Site 3	CCTGGAGTCTCTGCAGACTGA	GATGCAGGGACAAGGACAGA

Wt, wildtype; del, deletion; BS=binding site

Uncropped western blots

Fig.1A

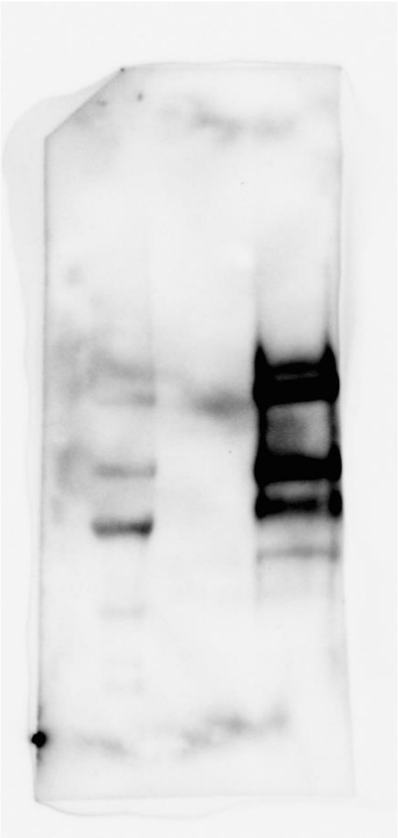

PDGFRA

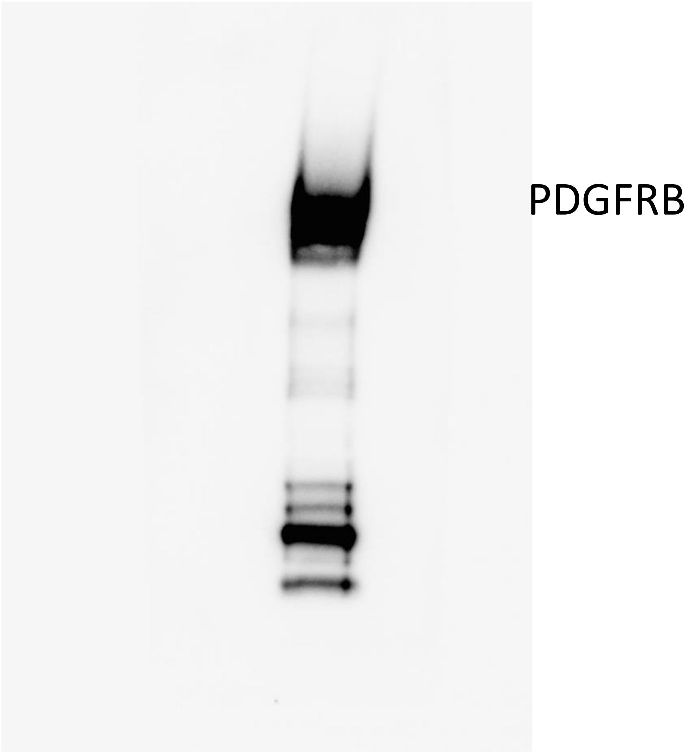

PDGFRB

Fig.1A

Tubulin

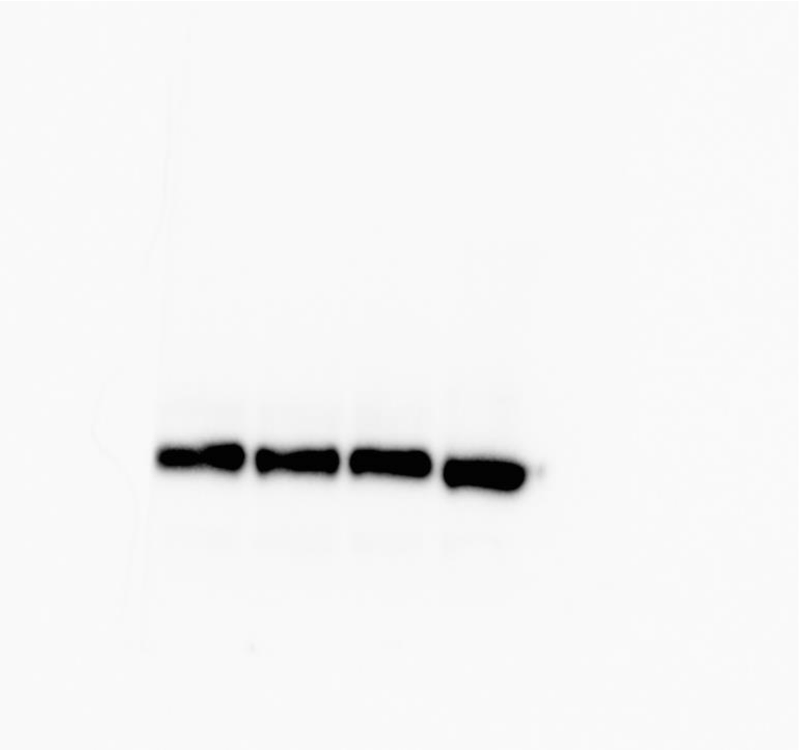

Fig.1E

SOS1 and b-actin

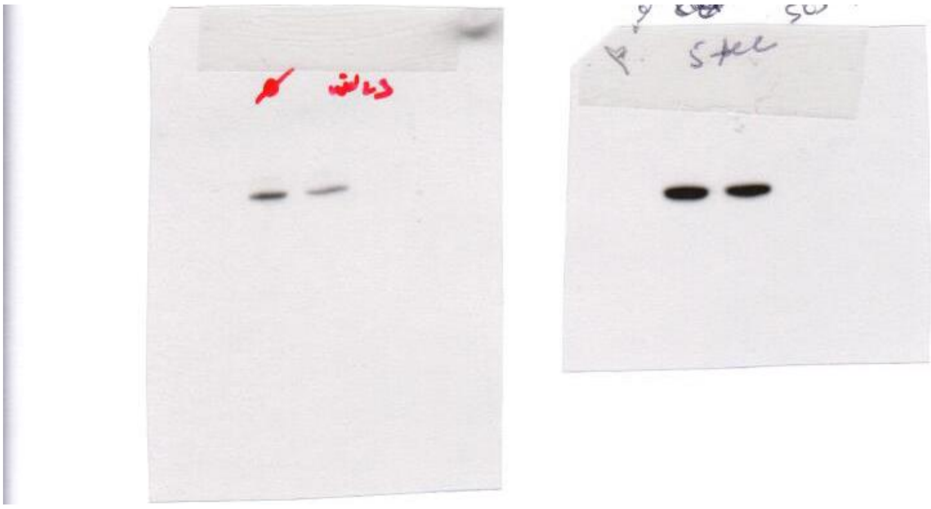

Fig.1E

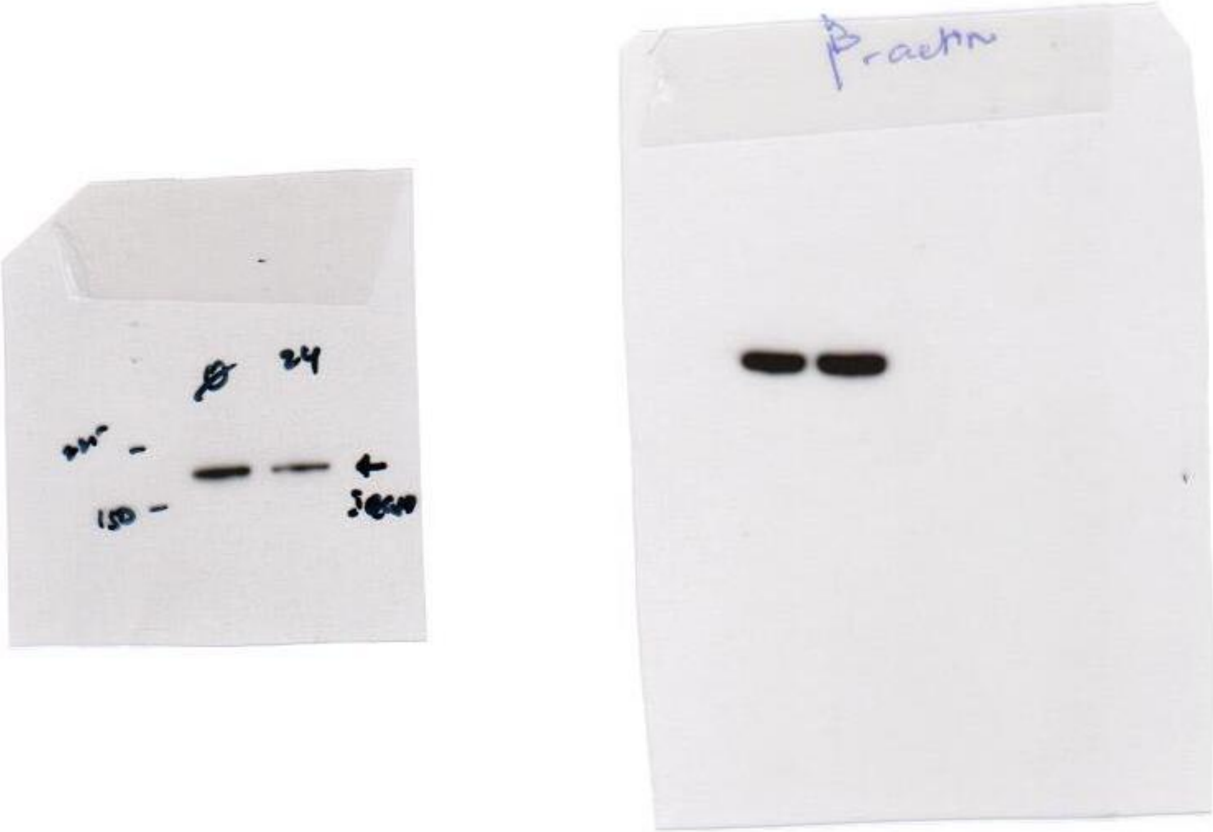

Fig.1E

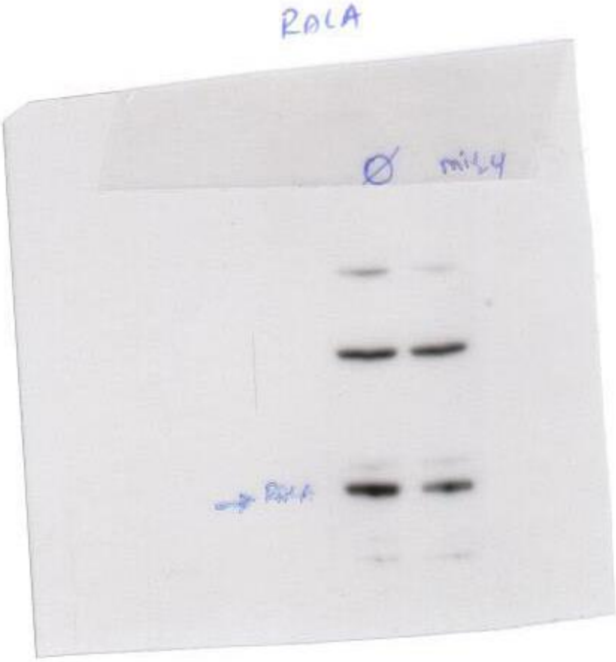

Fig.1E

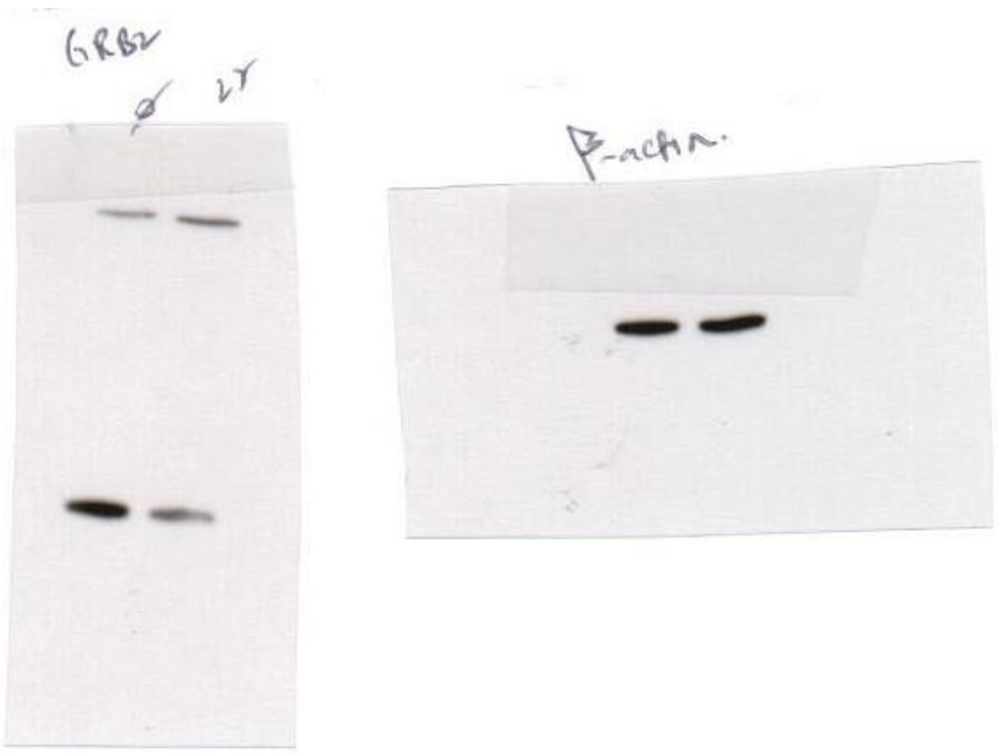

Fig.1E

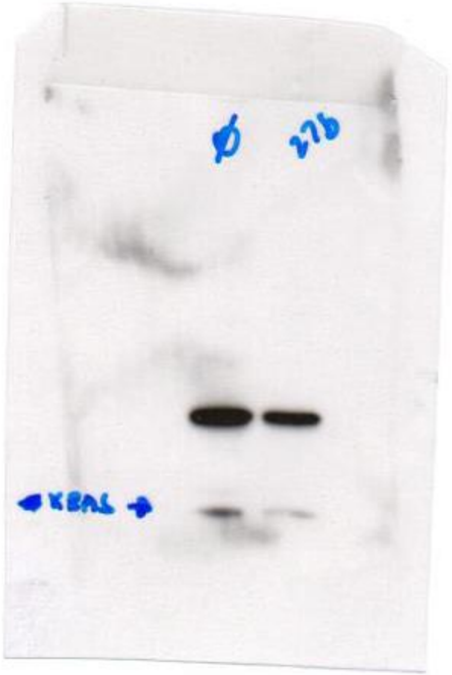

Fig.1E

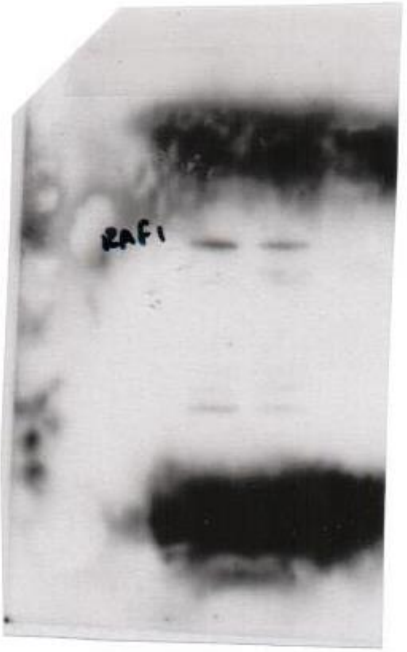

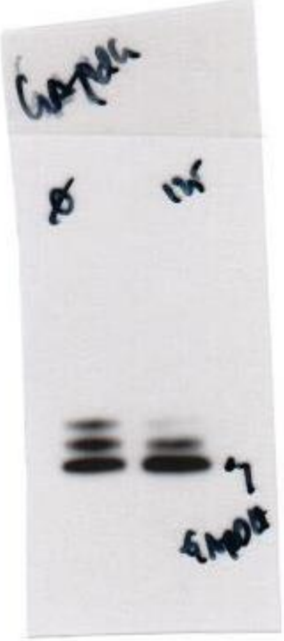

Fig.2A

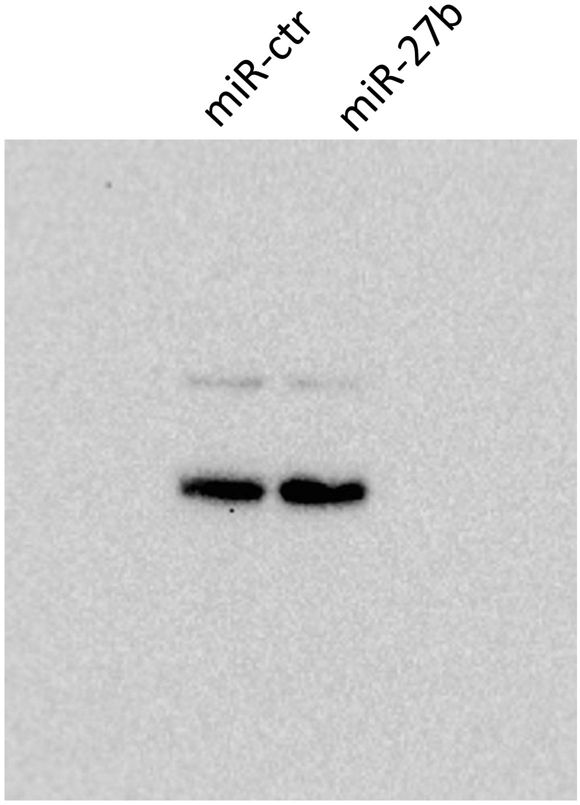

TOT AKT

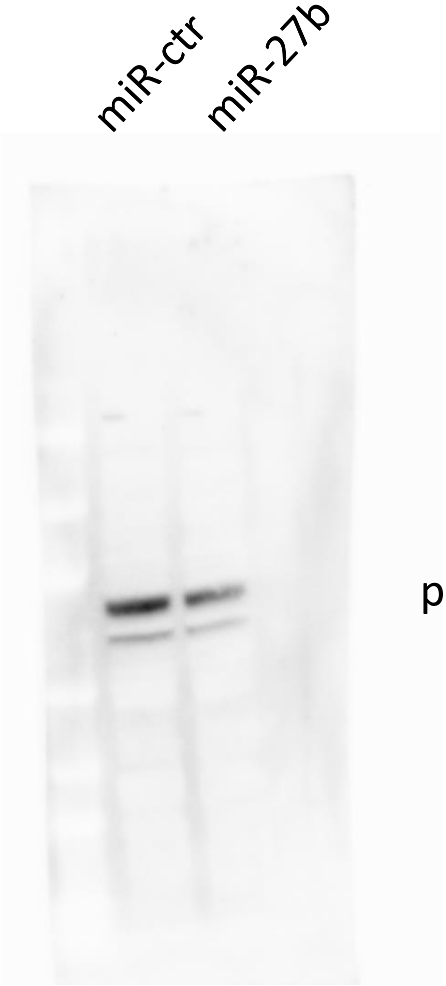

p-AKT

Fig.2A

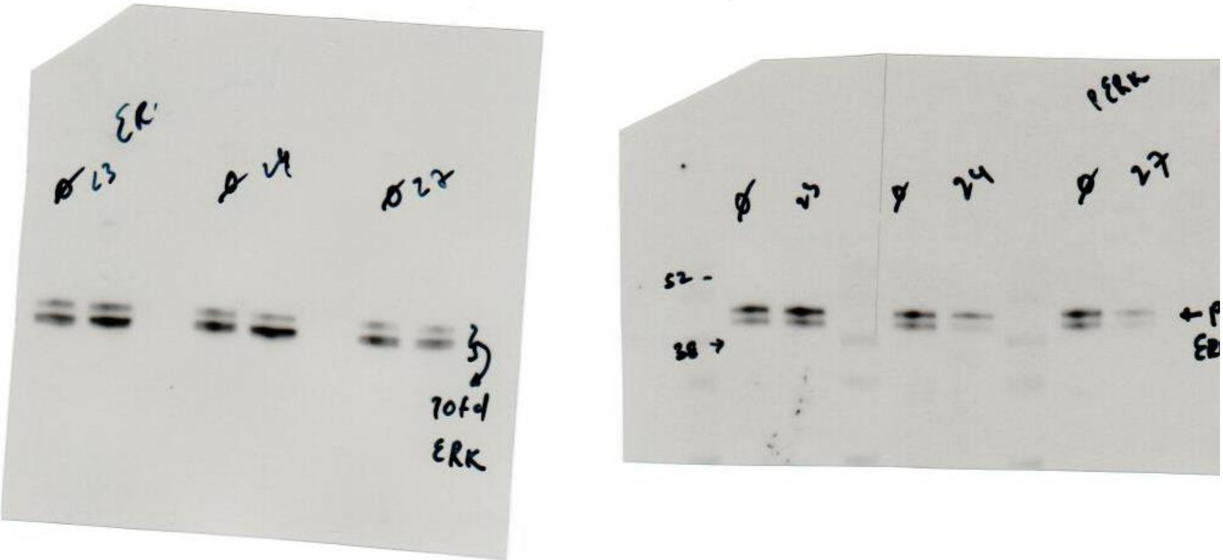

Fig.2A

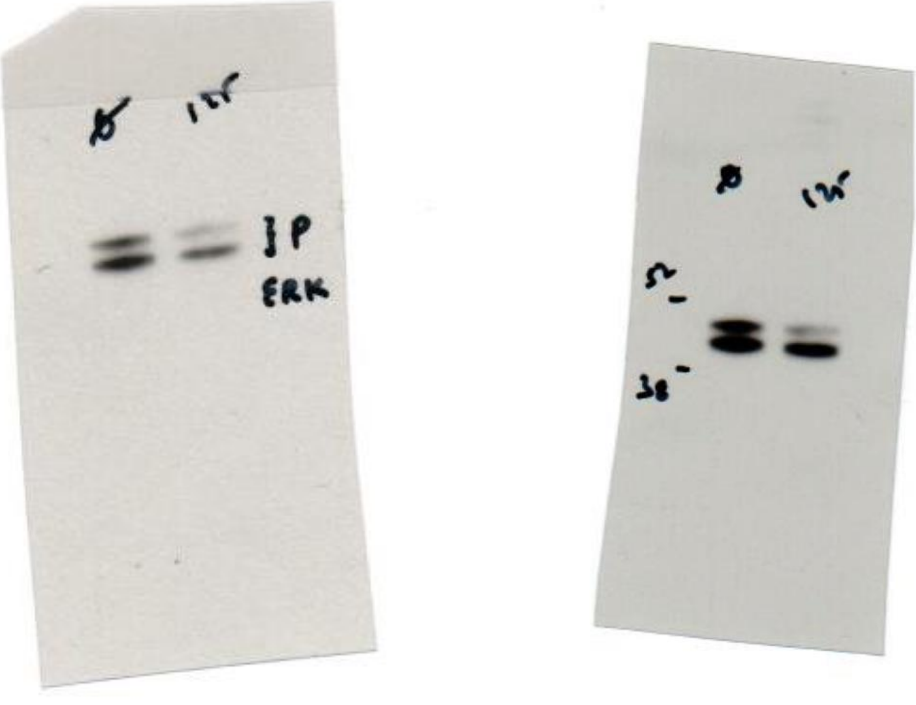

Tot ERKs

Fig.2A

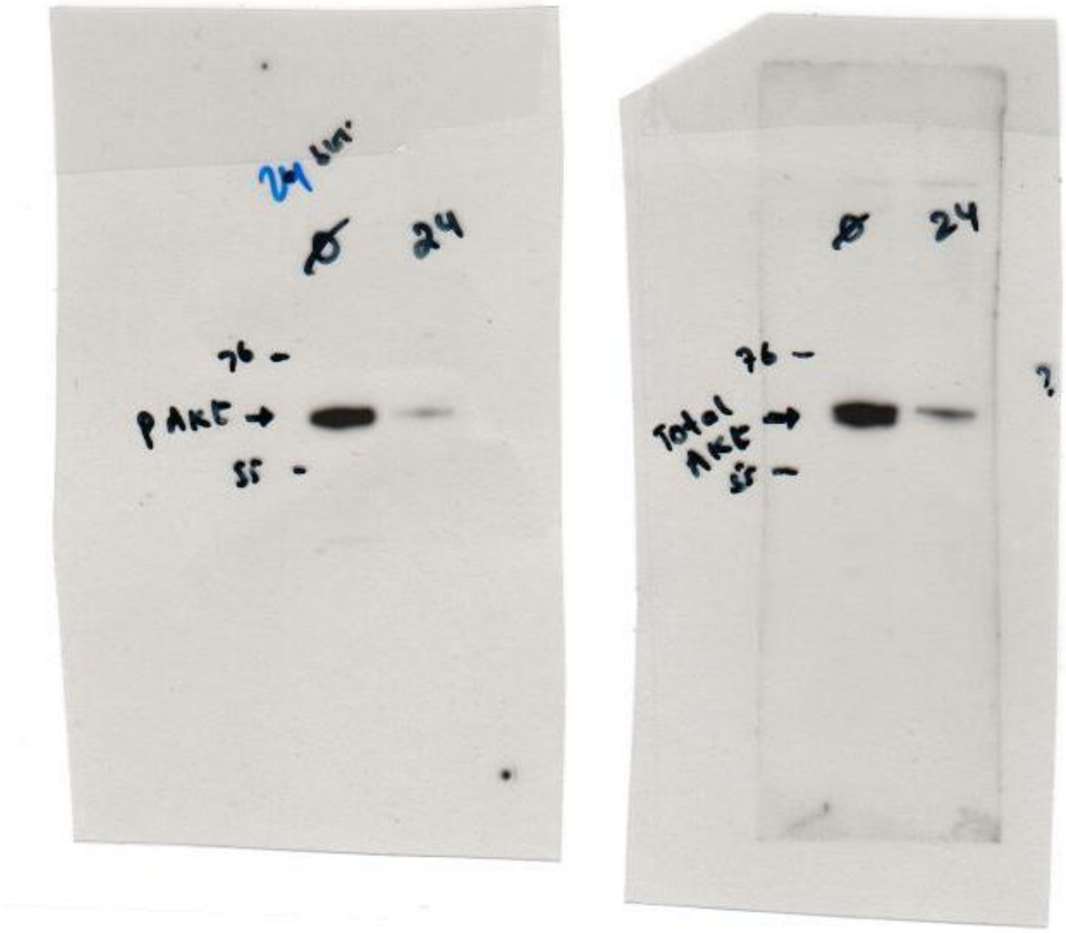

Fig.2D

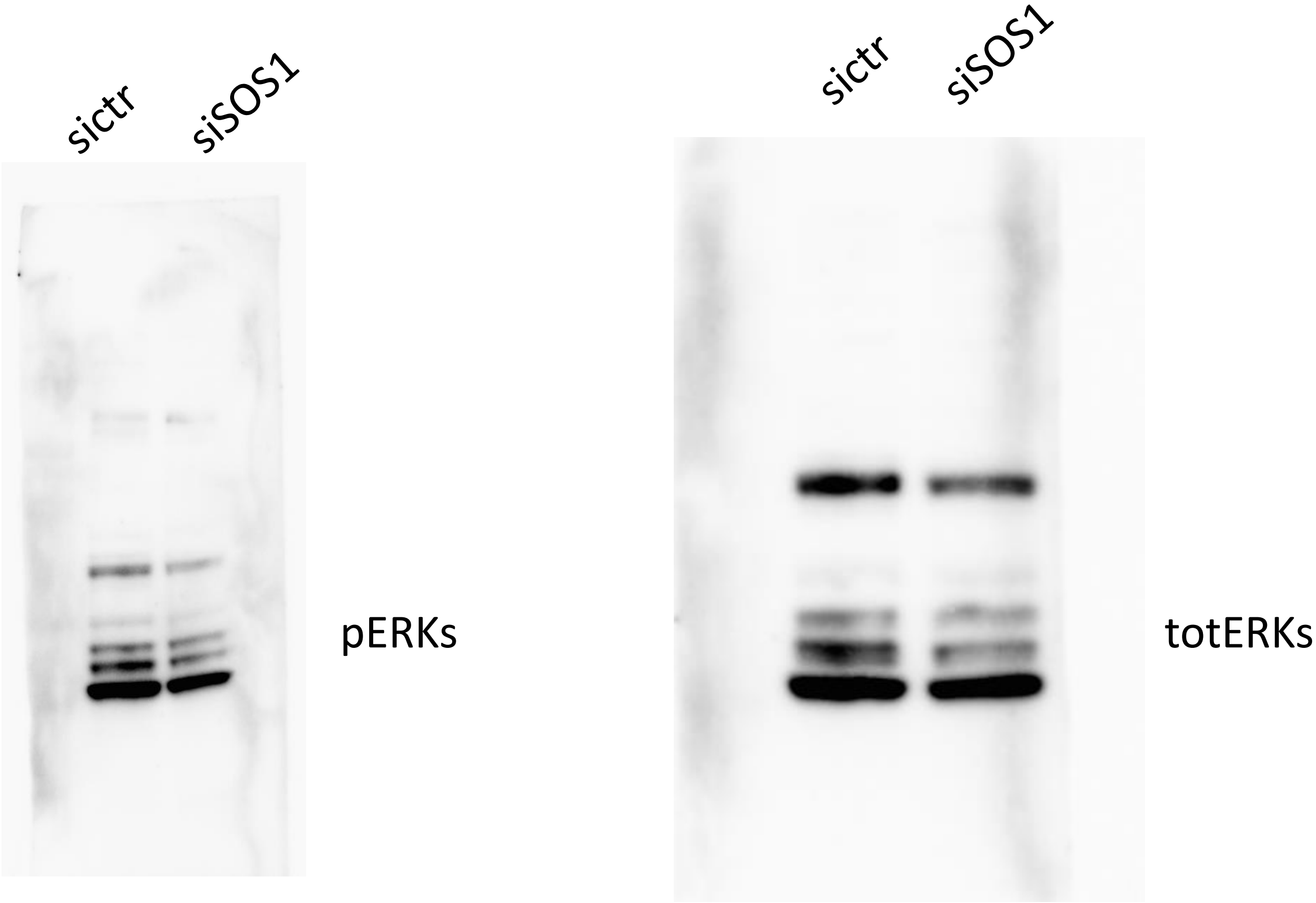

Fig.2A

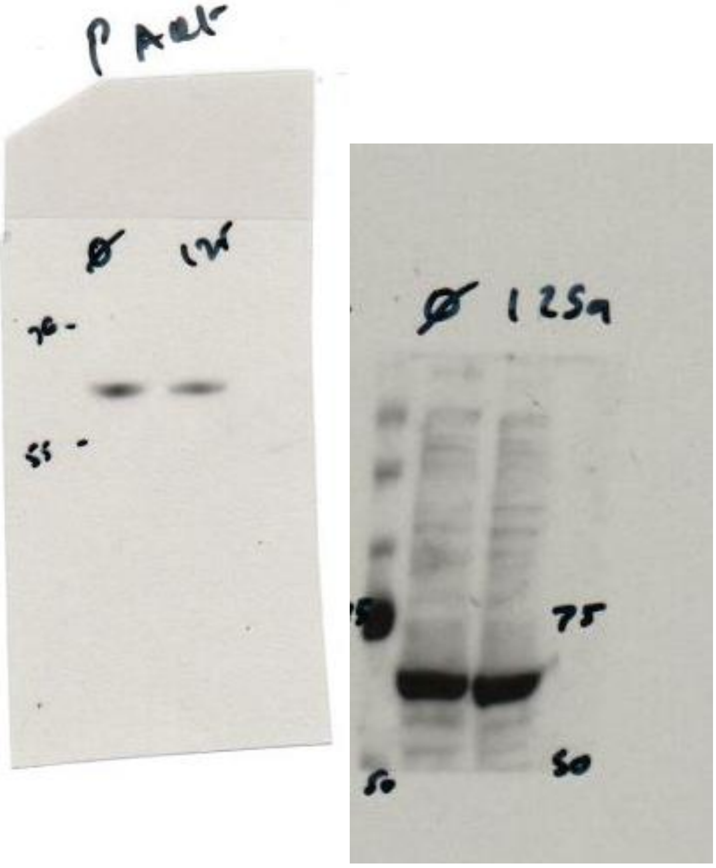

Tot AKT

Fig.2A

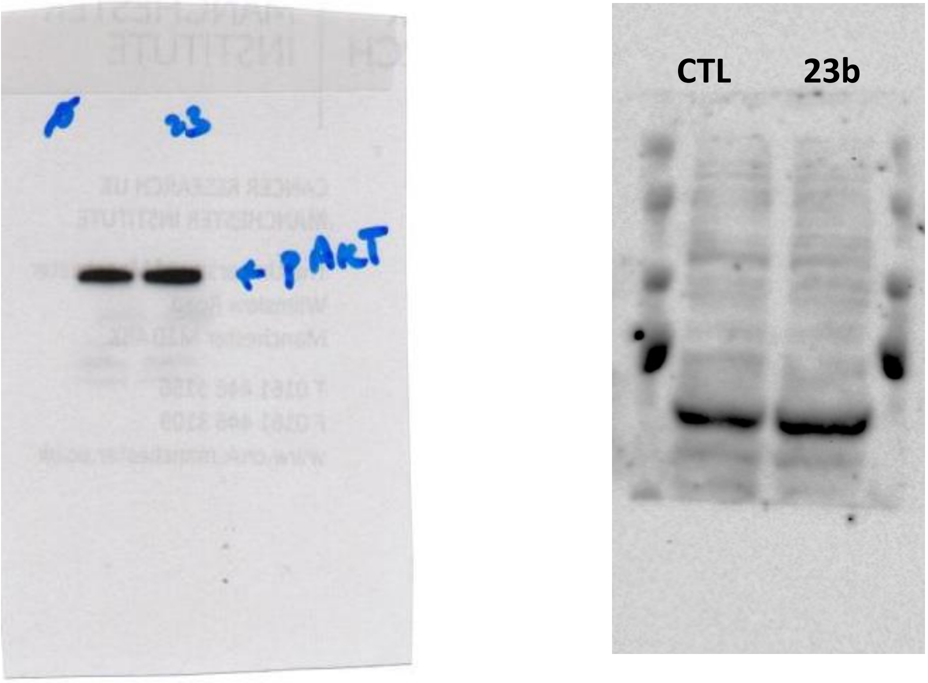

Tot akt

Fig.2B

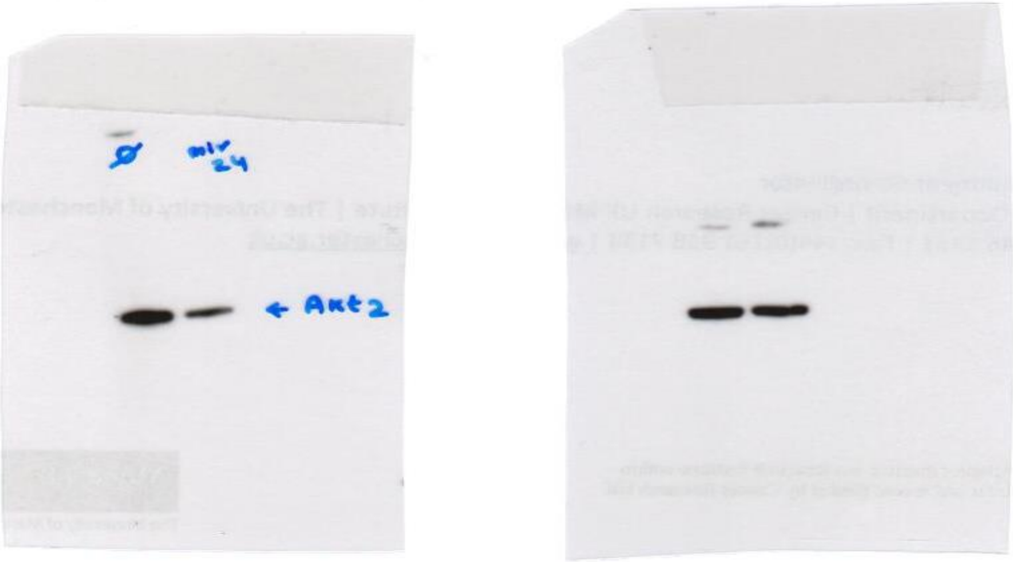

Fig.2B

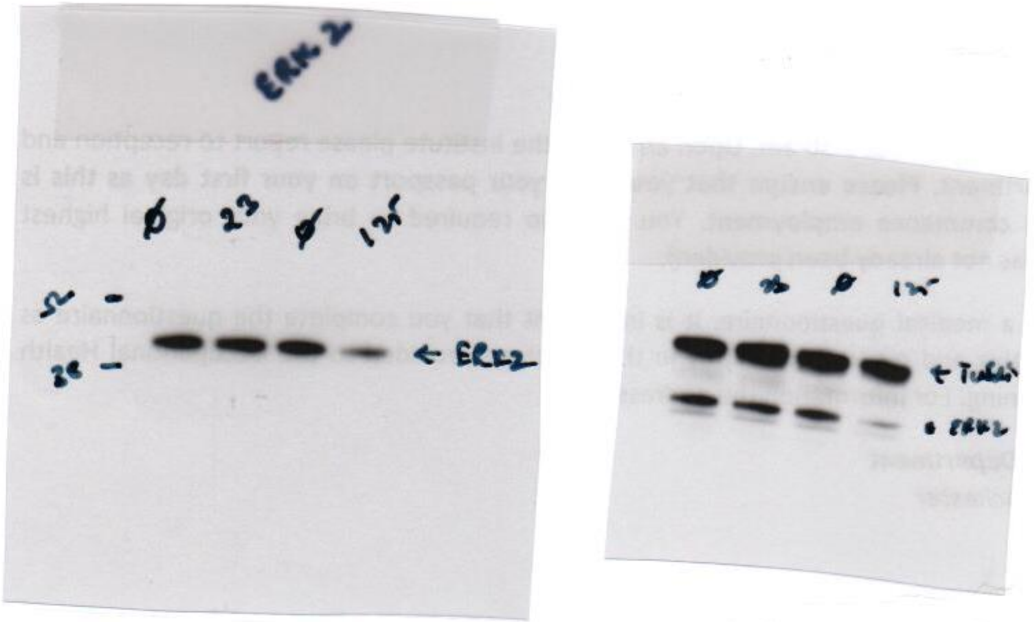

Fig.2D

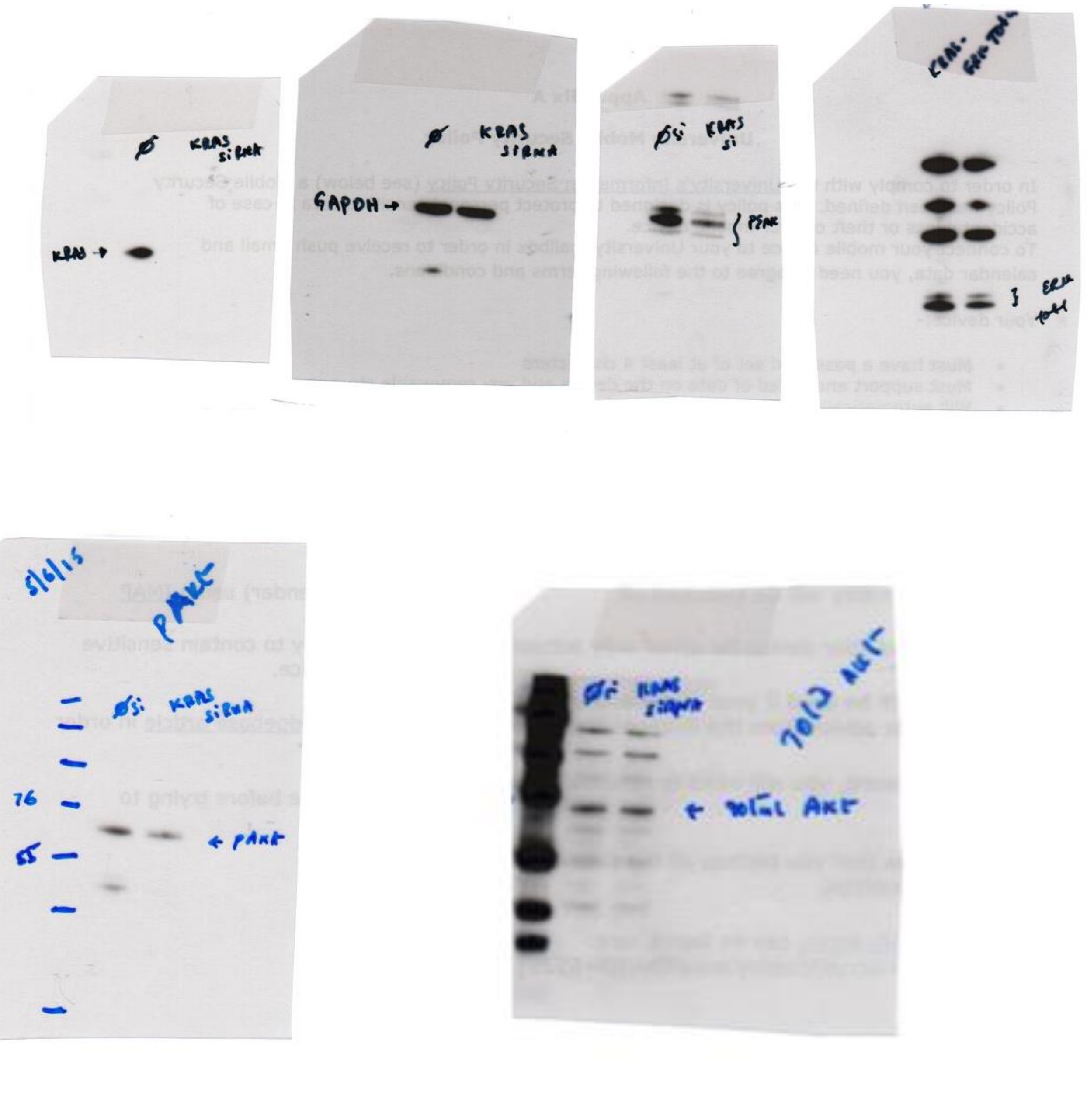

Fig.2D

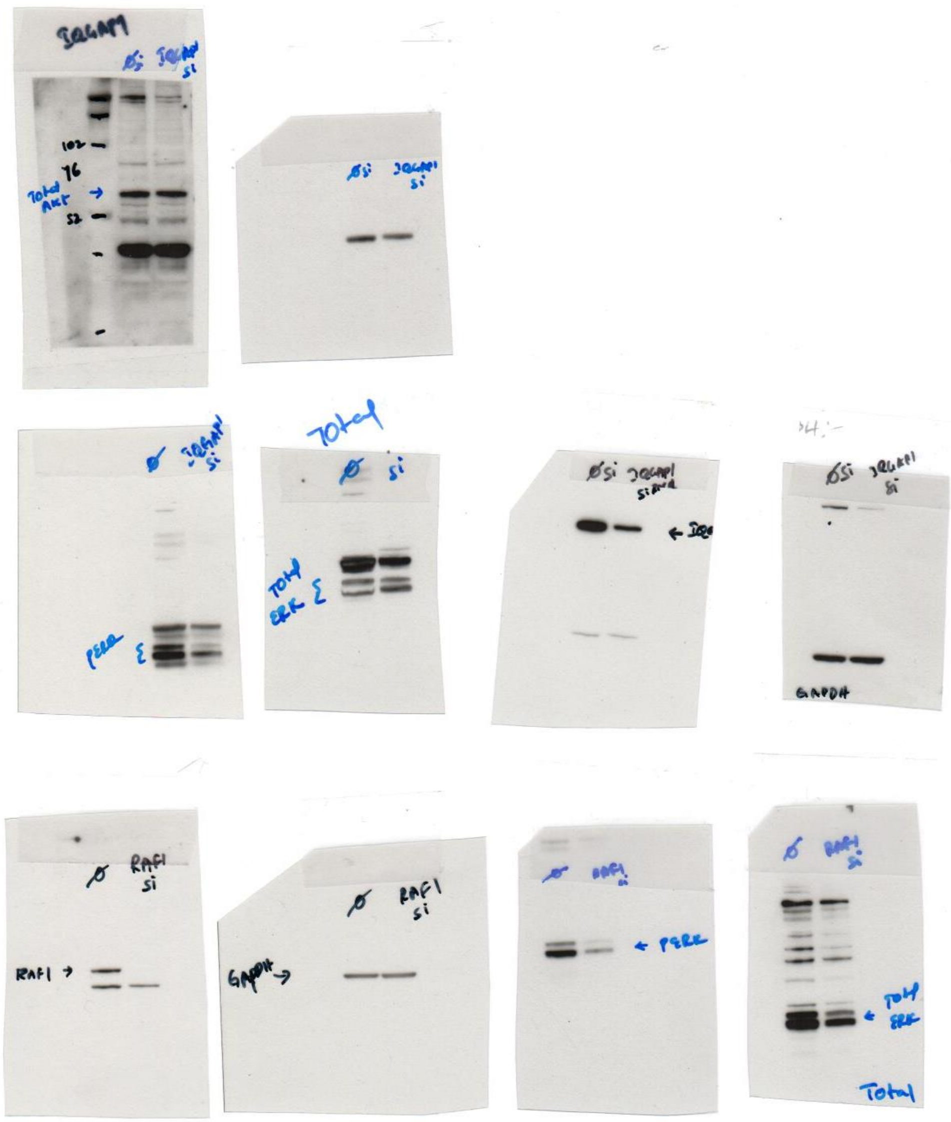

Fig.2D

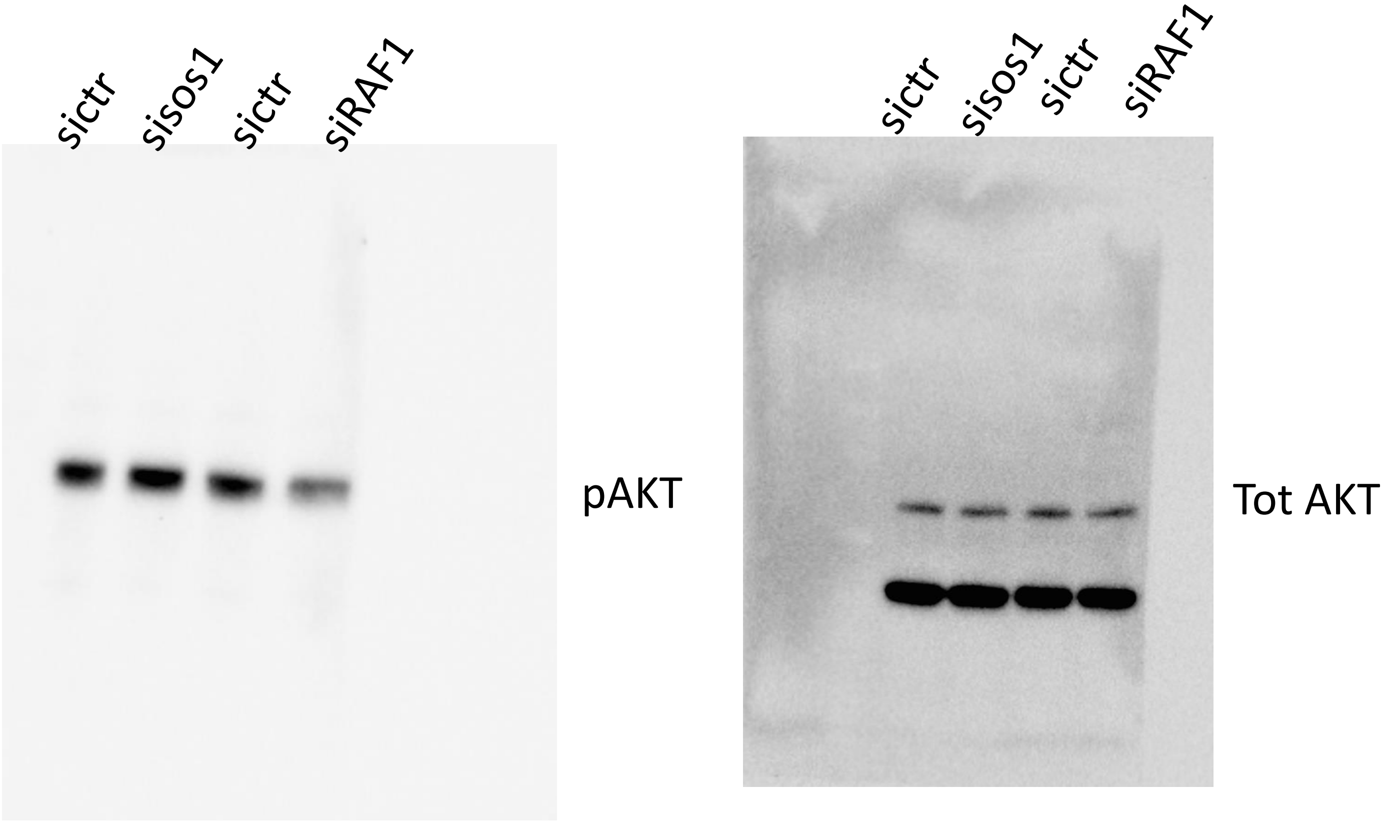
sictr
sisos1
sictr
siRAF1
pAKT
sictr
sisos1
sictr
siRAF1
Tot AKT

Fig.2D

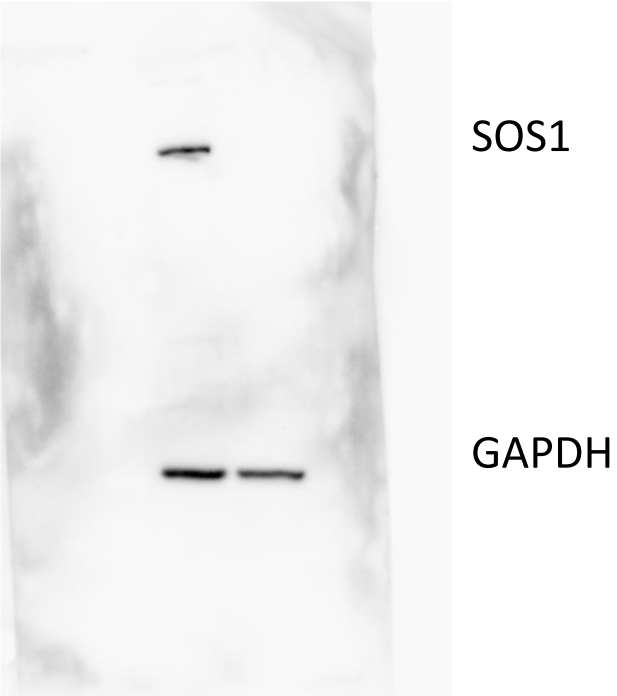
SOS1
GAPDH

Fig.4B

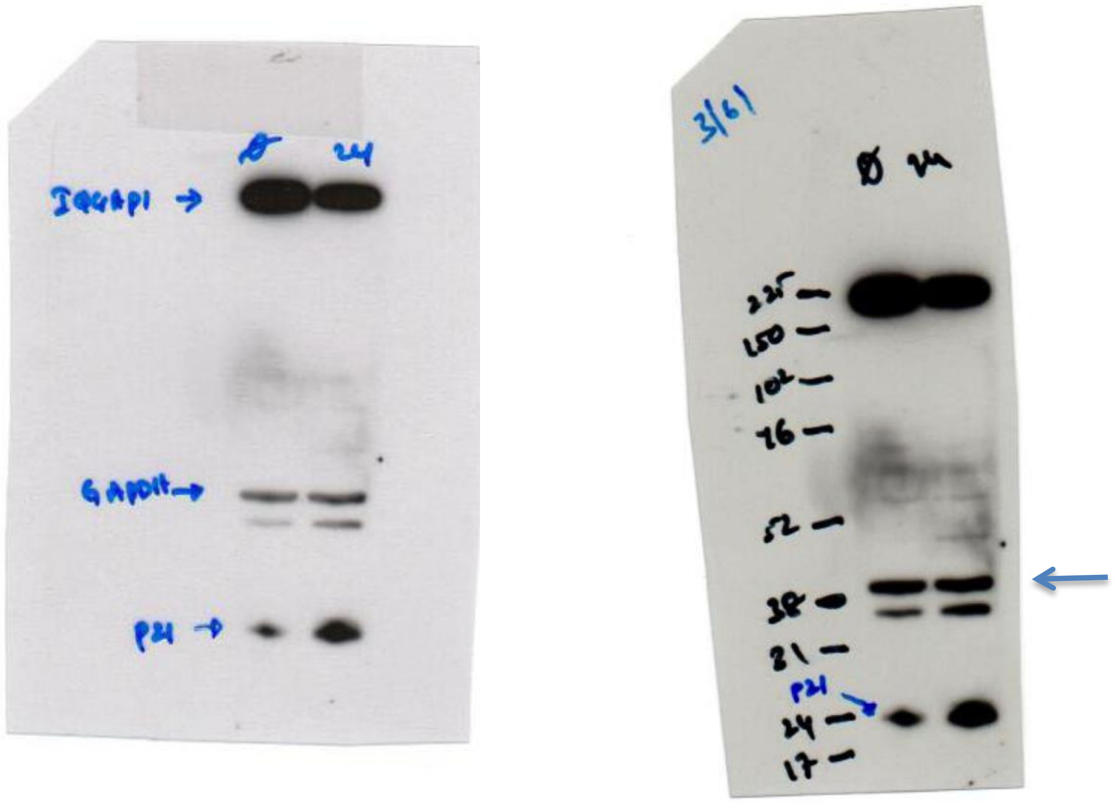

GAPDH

Fig.4B

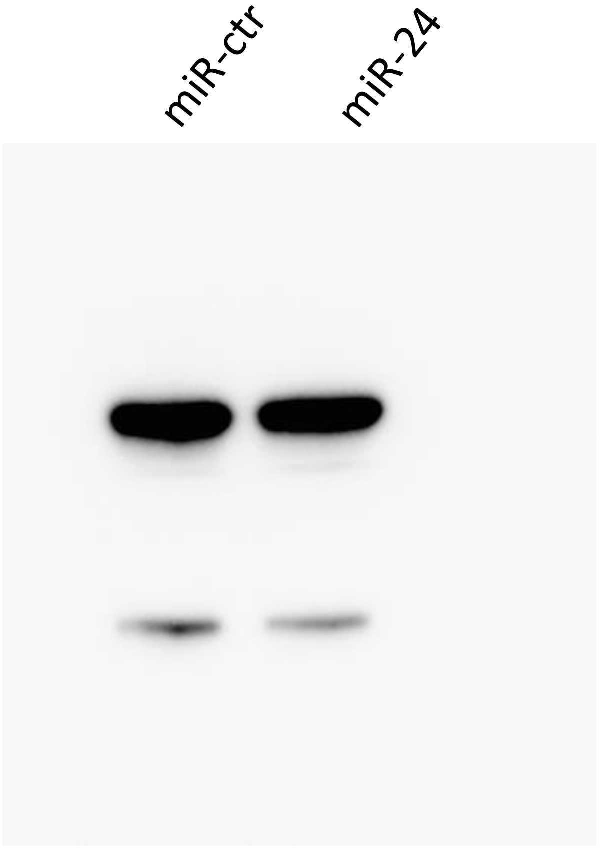

CDK1

Fig.5D

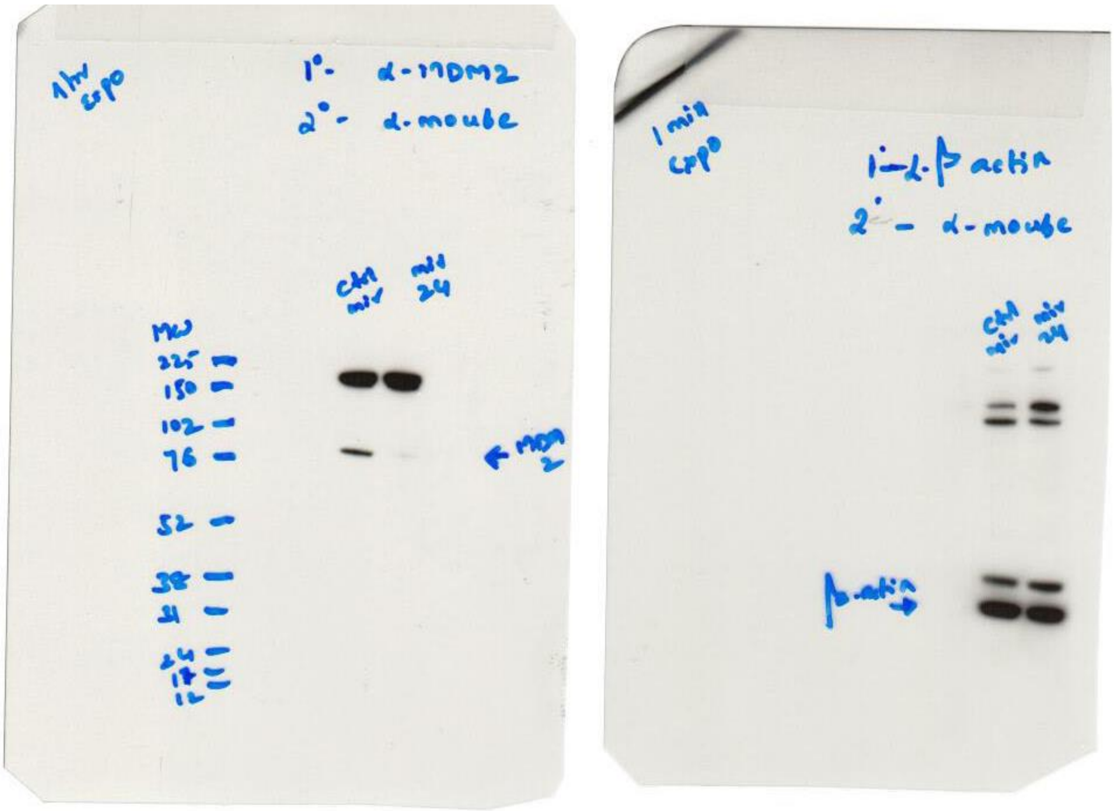

1° - α-MDM2
2° - α-mouse
MW
225
150
102
76
52
38
31
24
17
12
1° - α-β actin
2° - α-mouse
β-actin

Fig.5D

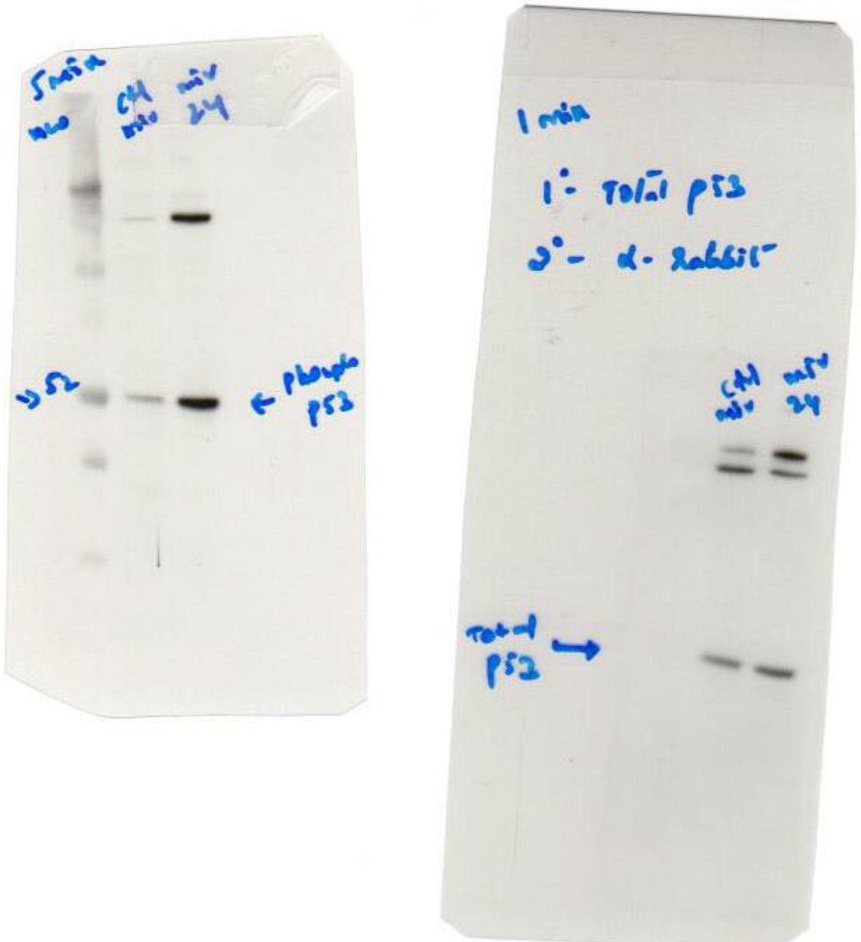

Fig.5E

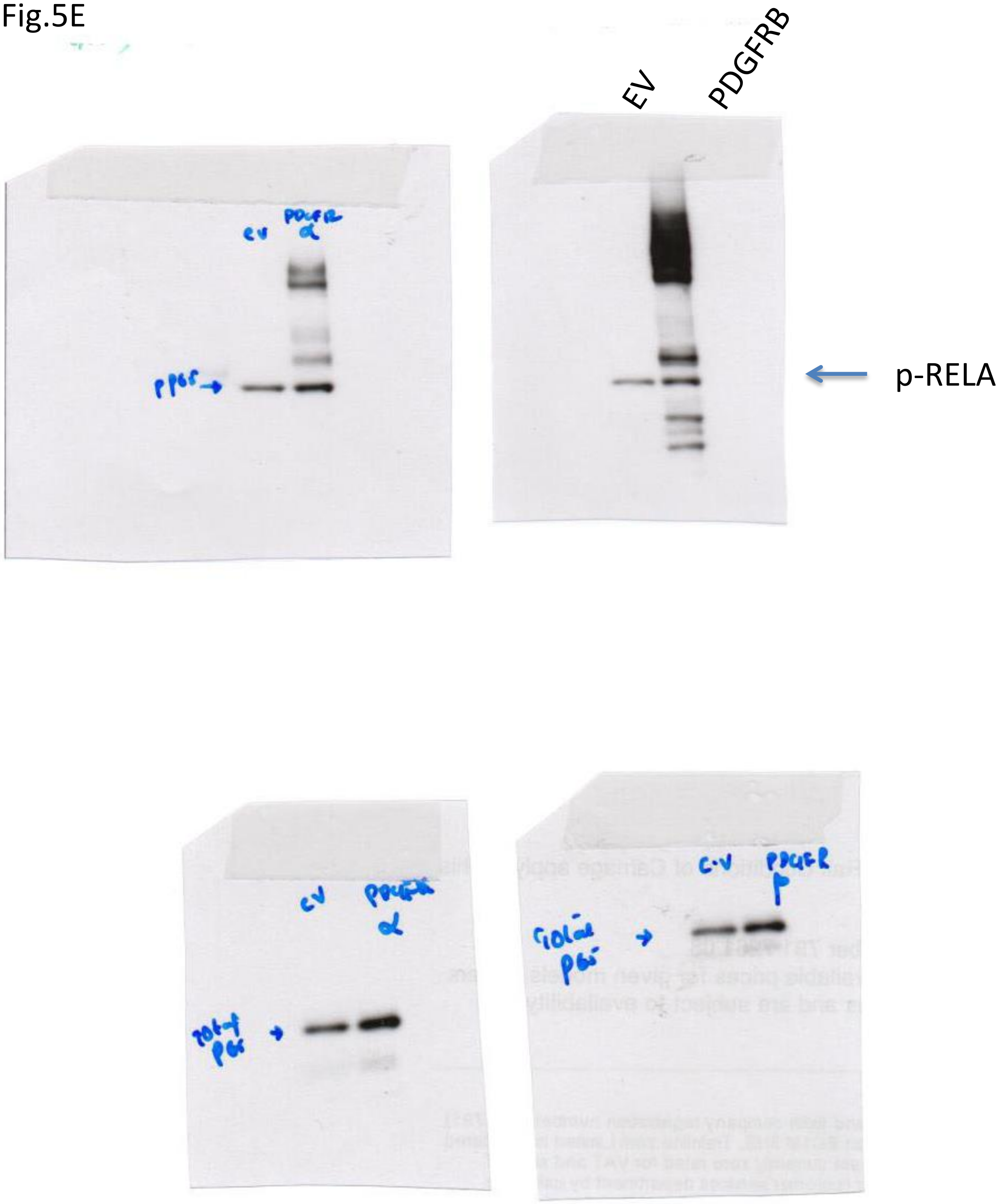

Fig.5E

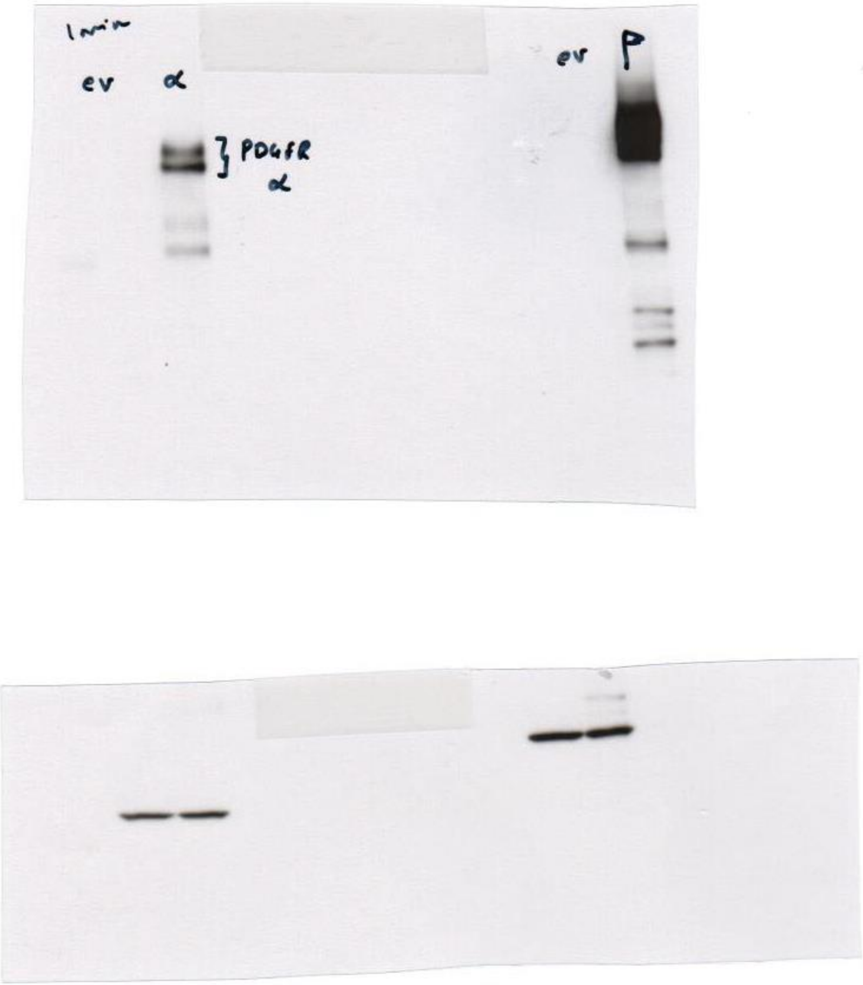

GAPDH

Fig.5H

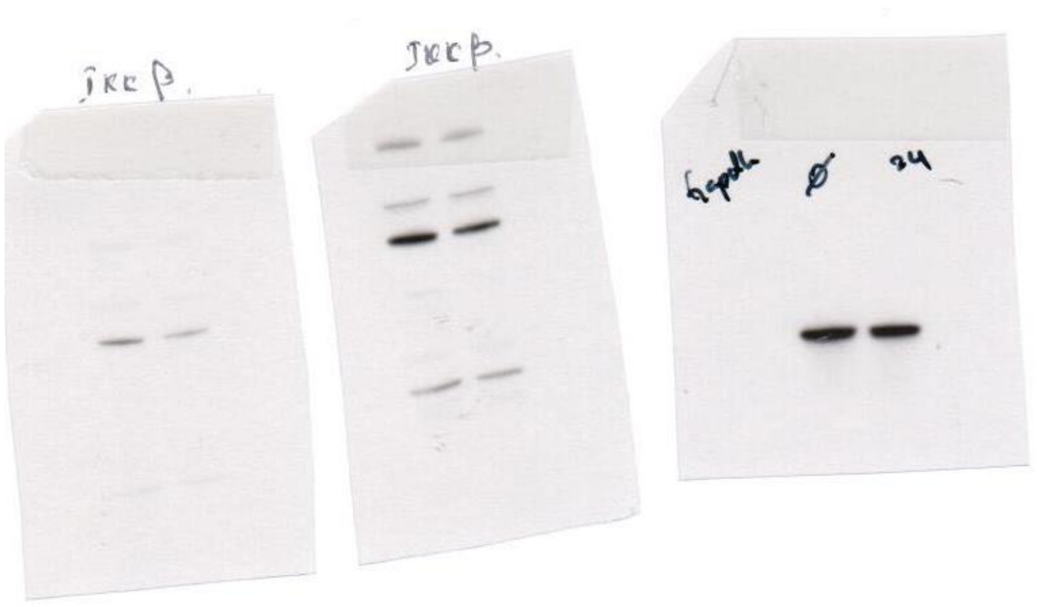

Fig.5I

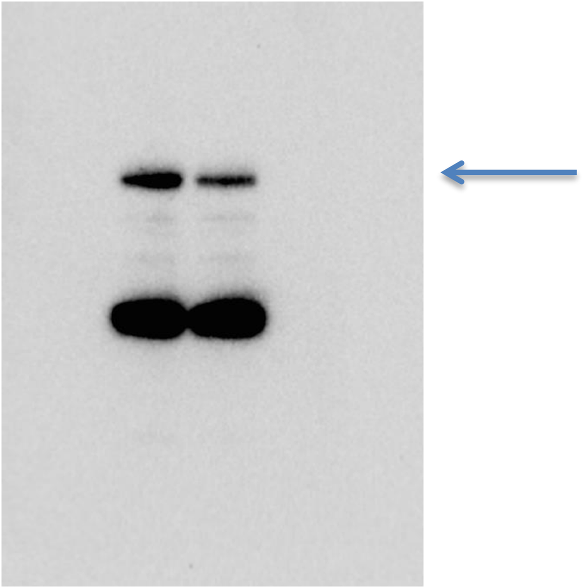

pRELA

Fig.5I

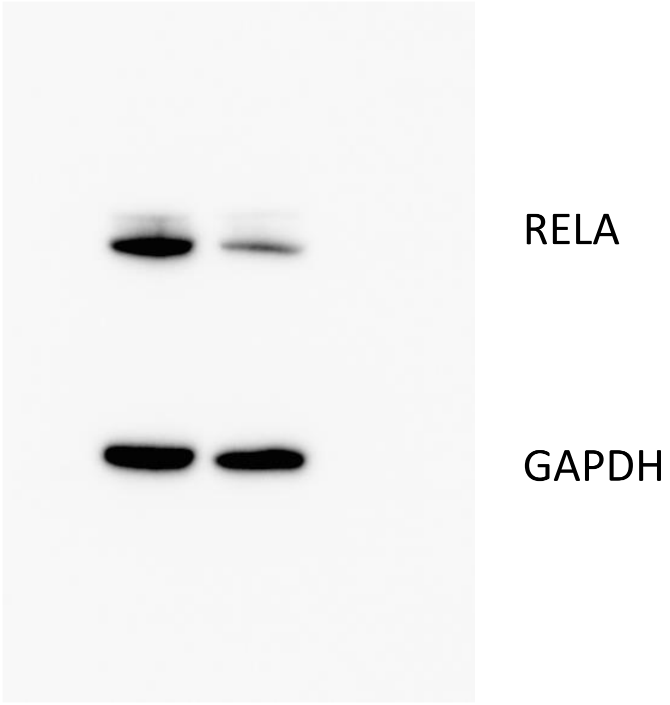

Suppl. Fig.1B

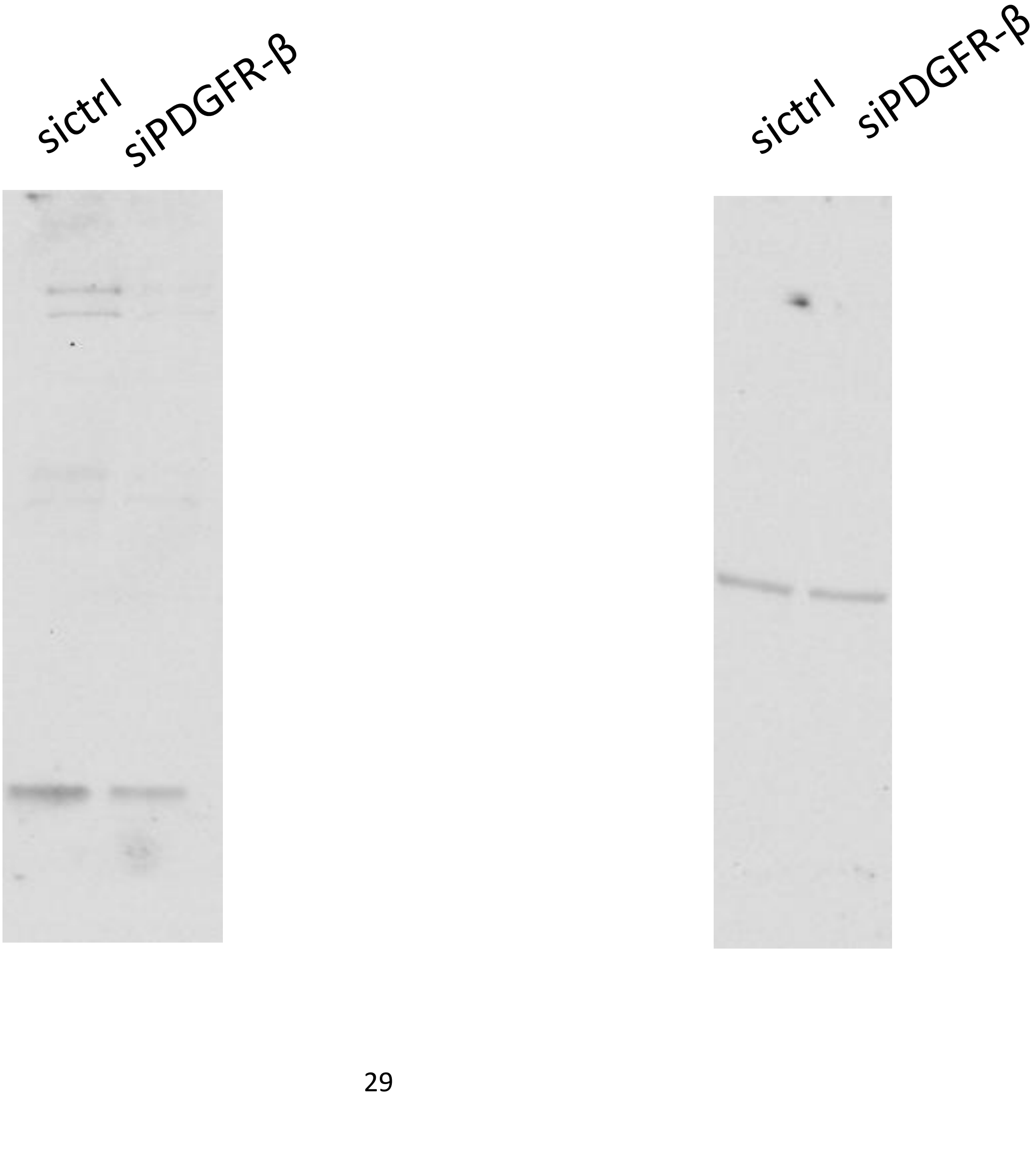

Suppl Fig. 3A

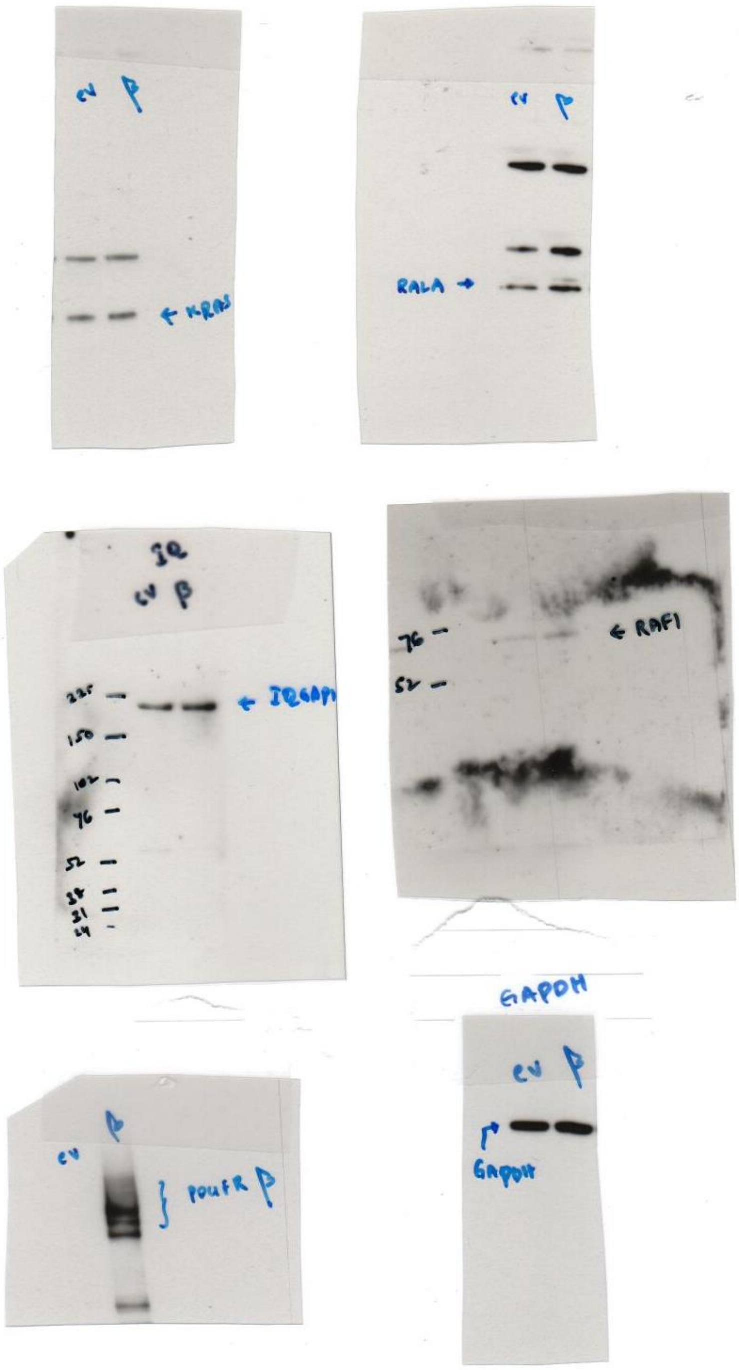

Suppl Fig. 3A

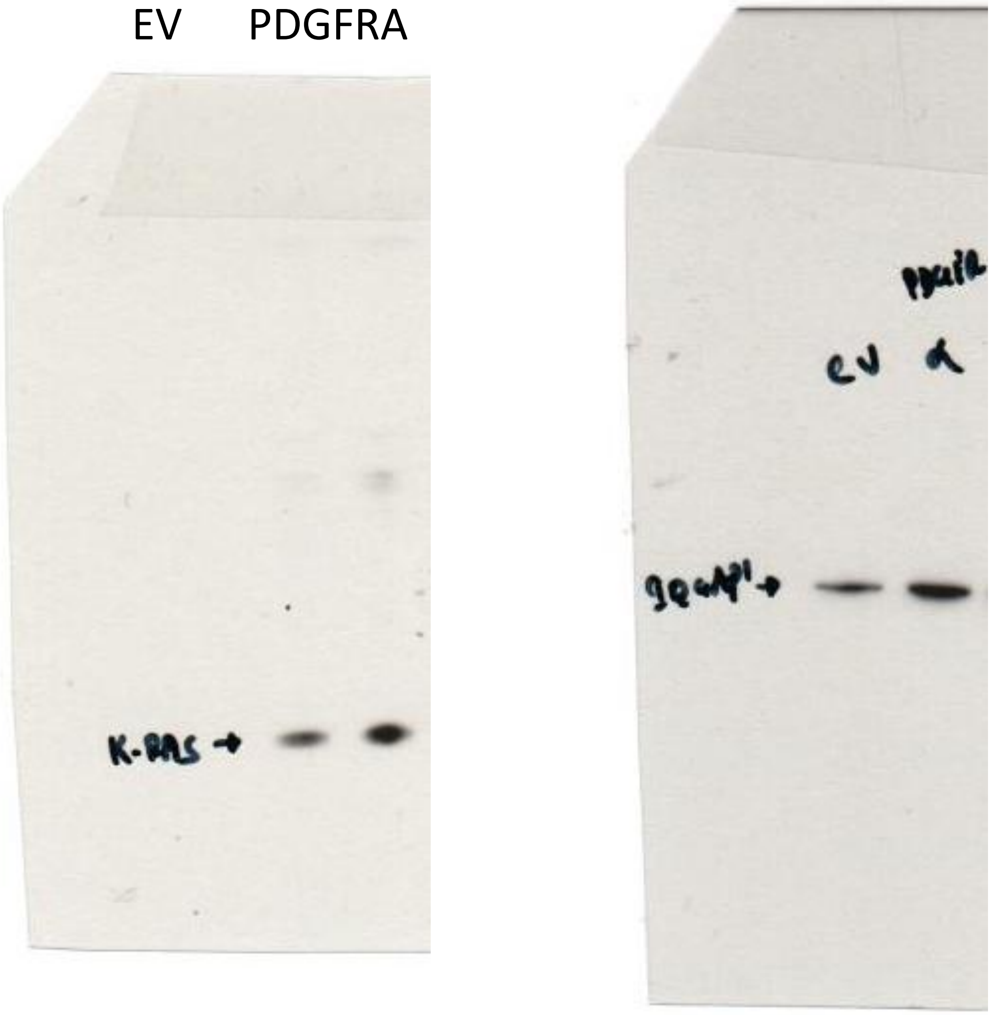

Suppl Fig. 3A

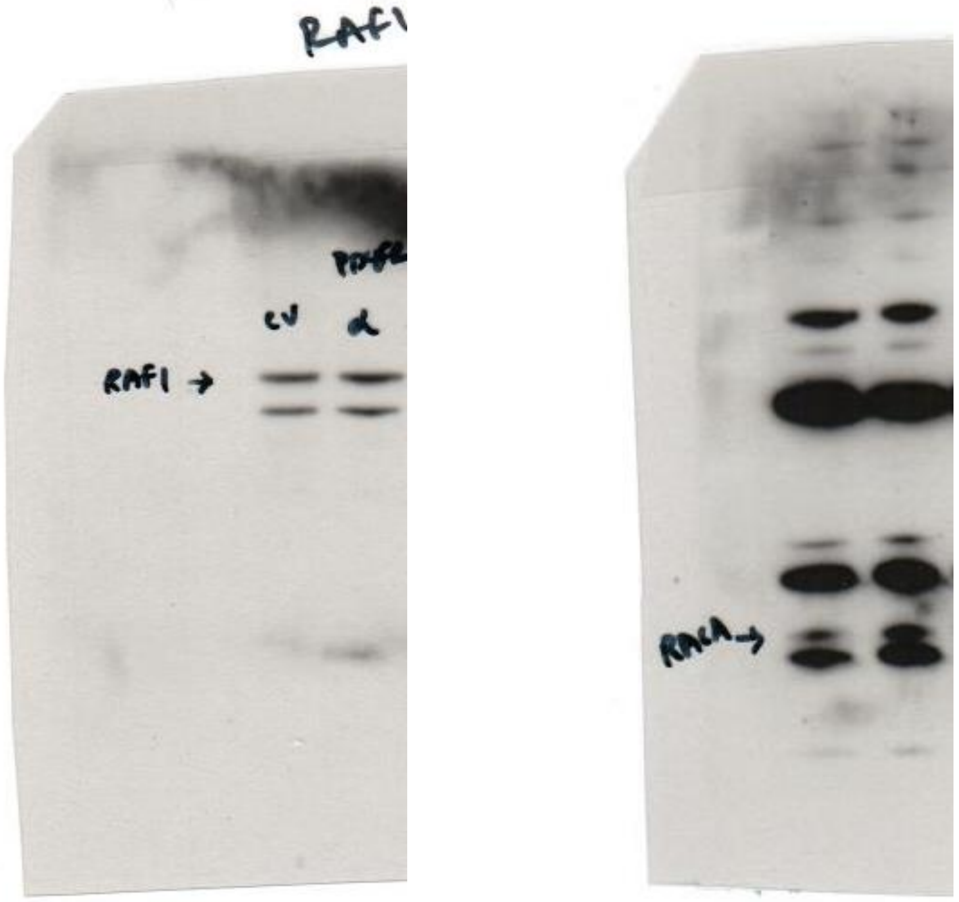

Suppl Fig. 3A

EV PDGFRA

GAPDH

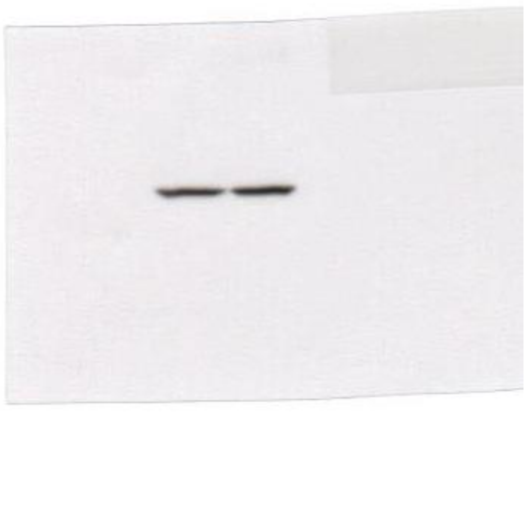

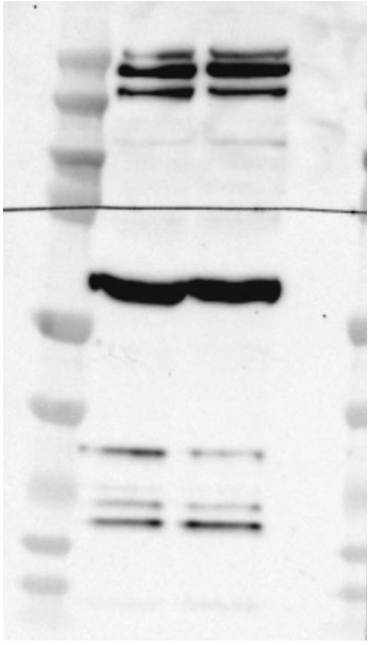

IQGAP1

RALA

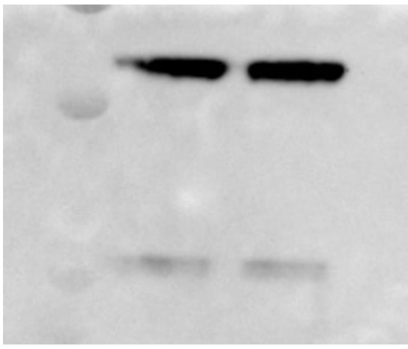

β-actin

KRAS

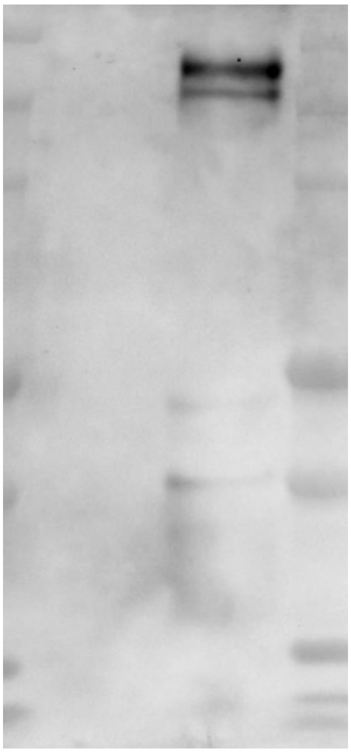

PDGFRB

Suppl. Fig.6A

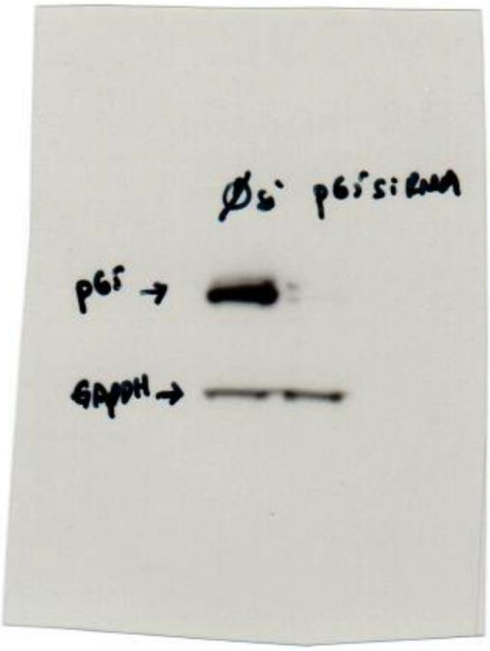

Supplement: Supplementary file 1 — Supplementary Information [file 41598_2017_14843_MOESM1_ESM.pdf]
